# 1st Global Consensus for Clinical Guidelines for the Rehabilitation of the Edentulous Maxilla: A Single‐Round Survey on Sinus Lift and Alveolar Bone Augmentation Techniques

**DOI:** 10.1111/clr.70018

**Published:** 2026-02-24

**Authors:** Giulia Brunello, Franz J. Strauss, Iva Milinkovic, Ina Kopp, Frank Schwarz, Hom‐Lay Wang

**Affiliations:** ^1^ Department of Oral Surgery University Hospital of Düsseldorf Düsseldorf Germany; ^2^ Department of Orthodontics and Dentofacial Orthopaedics Charité–Universitäts Medizin Berlin, Corporate Member of Freie Universität Berlin and Humboldt‐Universität Zu Berlin Berlin Germany; ^3^ Clinic of Reconstructive Dentistry Center of Dental Medicine, University of Zurich Zurich Switzerland; ^4^ Center for Studies and Innovation in Dentistry Faculty of Dentistry, Universidad Finis Terrae Santiago Chile; ^5^ Department of Periodontology and Oral Medicine and Implant‐Research Centre School of Dental Medicine, University of Belgrade Belgrade Serbia; ^6^ AWMF‐Institut für Medizinisches Wissensmanagement, Philipps‐Universität Marburg Marburg Germany; ^7^ Department of Oral Surgery, Implantology and Oral Medicine Goethe University Frankfurt am Main Germany; ^8^ Department of Periodontics & Oral Medicine University of Michigan School of Dentistry Ann Arbor Michigan USA

**Keywords:** bone grafting, bone regeneration, dental implants, maxillary sinus floor augmentation, sinus floor elevation

## Abstract

**Objectives:**

The objective of this survey study was to collect expert insights on the rehabilitation of the edentulous maxilla involving adjunctive procedures such as sinus lifts and other bone augmentation techniques. This process aimed to support the development of a consensus and contribute to the formulation of clinical practice guidelines for the management of the edentulous maxilla.

**Materials and Methods:**

In preparation for the 1st Global Consensus for Clinical Guidelines for the rehabilitation of the edentulous maxilla, a structured questionnaire was developed. The survey was administered online anonymously in a single round. It consisted of multiple‐choice items and 94 7‐point Likert‐scale statements and questions. Consensus was defined as >75% and ≤95% agreement or disagreement, and strong consensus as >95% agreement or disagreement.

**Results:**

Of the 217 experts invited from 43 countries, 116 successfully completed the questionnaire via the survey link, resulting in a response rate of 53.5%. Among the 94 statements and questions, 44 (46.8%) reached consensus and 4 (4.3%) strong consensus. All statements that achieved strong consensus pertained to key clinician‐reported outcomes considered essential for inclusion in future research. There was strong agreement on the importance of evaluating surgical, prosthetic, and biological complications, along with marginal bone loss.

**Conclusion:**

This study collected valuable expert opinions to inform a consensus development process and contribute to the formulation of clinical practice guidelines for the management of the edentulous maxilla in complex cases requiring additional augmentation procedures.

## 1 Introduction

Edentulism remains a significant global health issue, affecting over 300 million individuals worldwide (GBD 2021 Diseases and Injuries Collaborators 2024). The high prevalence of complete tooth loss highlights the urgent need for effective management and treatment strategies, given its profound impact on both oral and systemic health (Fagundes et al. 2024; Hunter et al. 2024; Linn et al. 2024). Tooth loss compromises chewing function, which can lead to malnutrition and adversely affects speech, appearance, self‐esteem, and social interactions, ultimately reducing quality of life (Fagundes et al. 2024).

While implant‐supported full‐arch prostheses have advanced in recent years (Messias et al. 2023), there is no universally accepted treatment strategy for rehabilitating the edentulous maxilla. This location presents unique challenges due to anatomical limitations and frequent requirements for additional surgical procedures such as sinus lifts and bone augmentation techniques (Avila‐Ortiz et al. 2023; Testori et al. 2024).

The choice of bone augmentation technique depends on the extent and configuration of the defect and could involve vertical, horizontal, or combined augmentation procedures. These can be performed either as staged procedures or simultaneously with implant placement (Aghaloo et al. 2016). A variety of grafting materials are available for bone augmentation, including autografts, allografts, xenografts, and synthetic bone substitutes, each offering specific advantages regarding biocompatibility, resorption rates, and osteoconductive properties (Raghoebar et al. 2019; Starch‐Jensen, Mordenfeld, et al. 2018). Additionally, there is increasing interest in the adjunctive use of blood derivatives (e.g., platelet‐rich fibrin) and growth factors (e.g., Bone morphogenetic protein 2, platelet‐derived growth factor) to accelerate the healing process (Calciolari et al. 2025; Galarraga‐Vinueza et al. 2024; Jung et al. 2003; Miron et al. 2024; Strauss et al. 2020; Strauss et al. 2018; Thoma, Payer, et al. 2018; Valentini et al. 2025). However, current clinical evidence supporting these approaches remains limited.

Sinus lift, also known as maxillary sinus floor augmentation, is often necessary to address the insufficient bone height in the posterior maxilla. These procedures are typically performed using either the lateral window technique or a crestal approach, depending on the residual bone height (Lyu et al. 2023). Both approaches aim to elevate the Schneiderian membrane to enable dental implant placement either with or without the use of grafting materials. While the lateral window technique, first described by Tatum in the 1980s, remains the most widely used approach, the crestal technique has gained popularity in selected indications for its reduced invasiveness (Raghoebar et al. 2019).

Advances in diagnostic imaging, such as cone‐beam computed tomography (CBCT), have greatly improved preoperative planning by allowing more accurate assessment of bone volume and quality (Morgan et al. 2023). Furthermore, guided surgery techniques can enhance the accuracy of prosthetically‐driven implant positioning (D'Haese et al. 2017). Despite these advances, treatment approaches for the edentulous maxilla continue to vary, and a lack of standardized, evidence‐based protocols persists.

In response to this need for clarity and standardization, the 1st Global Consensus for Clinical Guidelines (GCCG) 2025 was established. This initiative, titled “Patient‐Centered Clinical Workflow in Implant Dentistry,” brought together leading international experts to develop evidence‐based, patient‐centered guidelines for the rehabilitation of the edentulous maxilla. As part of this effort, a single‐round survey was conducted, to systematically gather expert opinions on implant‐supported maxillary rehabilitations. The findings were evaluated during the 1st GCCG Workshop alongside existing evidence, gathered through systematic reviews prepared as an integral part of the GCCG preparation, to inform the consensus development process, identify gaps in knowledge and pinpoint areas requiring further systematic reviews or research with the ultimate aim of improving clinical protocols and enhancing patient care.

The present summary focuses on the responses that specifically addressed maxillary rehabilitations in cases requiring additional procedures such as sinus lifts and other bone augmentation techniques to enable prosthetically driven implant placement.

## 2 Materials and Methods

The protocol for the present study received approval from the Ethical Committee at the University of Düsseldorf (Protocol no. 2024‐2973). The study was carried out and documented in accordance with the guidelines outlined in the “Good Practice in the Conduct and Reporting of Survey Research” (Kelley et al. 2003).

### 2.1 Study Design

This study was conducted as a single‐round survey in anticipation of the GCCG workshop, which was set to be held in Boston in June 2025. The aim of this study was to investigate emerging trends and recent advancements in the rehabilitation of the edentulous maxilla, specifically focusing on sinus lift and bone augmentation techniques. Additionally, the study sought to address the gap between common clinical approaches and the available literature, highlighting areas that require further exploration and uncovering discrepancies between the perspectives of professionals in the field and the current body of scientific evidence.

The results of this survey constituted the basis for identifying key questions to be explored in subsequent systematic reviews, further research or discussed directly during the GCCG workshop. The Scientific Chairs of the GCCG (Frank Schwarz, Hom‐Lay Wang), supported by the Survey Panel (Giulia Brunello, Todd Schoenbaum) and the Cross‐disciplinary Expert and Patient Panel (Franz‐Josef Strauss, Guo‐Hao Lin), finalized the questionnaire and coordinated the process of expert selection and invitation.

### 2.2 Questionnaire

The questions for experts were formulated based on the existing literature, focusing on the rehabilitation of the edentulous maxilla through sinus lift and bone augmentation techniques. The initial draft of the questionnaire was created by the Survey Panel, who played a key role in framing the initial set of questions.

To guarantee the questionnaire's clarity and relevance, it underwent an iterative validation process, consisting of multiple rounds of feedback and revisions. In addition to the Scientific Chairs, contributors included the Survey Panel and the Cross‐disciplinary Expert and Patient Panel. Their input facilitated the refinement of the questionnaire before ethical approval.

The questionnaire was developed in English and intended to be completed within 20–30 min. The final version, which includes 33 items (see Appendix), was structured as follows:

- declaration of consent (item 1);
- professional specialization and working environment (items 2–3);
- planning phase (items 4–7);
- guided surgery (item 8);
- timing of implant placement and loading (items 9–11);
- biomaterials and biologics (e.g., blood concentrates) (item 12–14, 16);
- provisional restorations (item 15);
- antibiotic prescription in relation to implant placement (items 17–21);
- maintenance (item 22);
- future trends (item 23);
- Schneiderian membrane perforation (items 24–26);
- soft tissue augmentation (item 27);
- implant longevity (item 28);
- factors affecting patient satisfaction (item 29);
- factors influencing selection of treatment procedures (items 30–31);
- fundamental outcomes to be included in future studies (items 32–33).

### 2.3 Expert Selection and Data Collection

**FIGURE 1:**
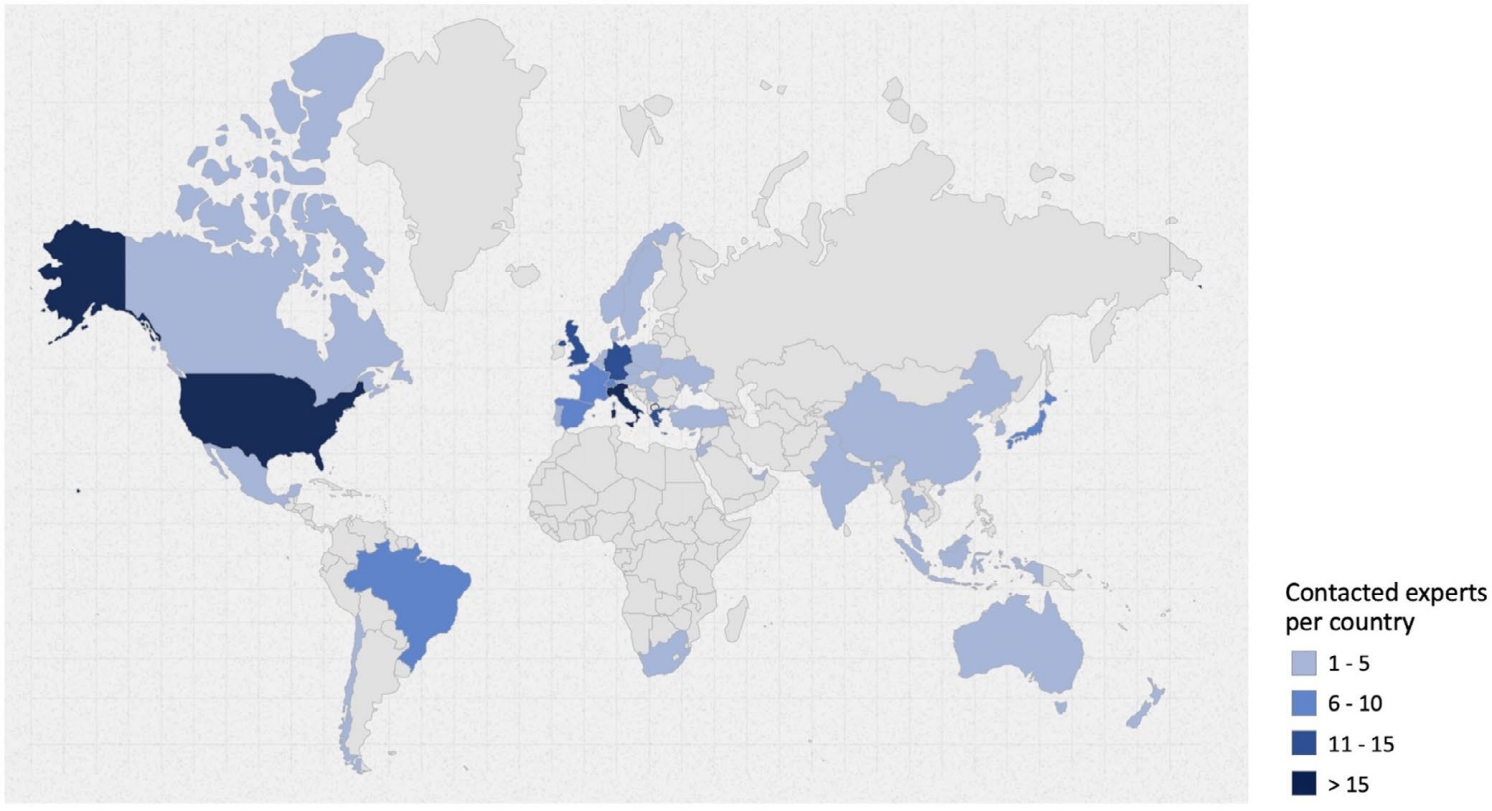
Distribution by country of the contacted experts. Map created using the open source online tool available at https://www.amcharts.com/ (amCharts, Neringa, Lithuania).

There is no standardized formula for determining the ideal number of participants in a survey study in the context of a consensus conference, with sample size recommendations generally relying on the COMET Initiative guidelines (Williamson et al. 2017) and previous similar projects (Alarcon et al. 2021; Madianos et al. 2016; Sanz et al. 2019). Thus, a target sample size of 100 experts was considered adequate. To account for an anticipated response rate of 50%, 217 experts from 43 countries were selected and invited to participate in the survey (Table 1; Figure 1).

**TABLE 1:** Details and distribution by country of the contacted experts.

Country	No.	%
Australia	4	1.8
Austria	2	0.9
Belgium	5	2.3
Brazil	9	4.1
Canada	5	2.3
Chile	2	0.9
China	5	2.3
Cyprus	2	0.9
Czech Republic	1	0.5
Denmark	3	1.4
France	7	3.2
Germany	15	6.9
Greece	11	5.1
Hungary	1	0.5
India	1	0.5
Indonesia	1	0.5
Israel	2	0.9
Italy	20	9.2
Japan	6	2.8
Jordan	4	1.8
Korea (Republic of)	1	0.5
Lebanon	3	1.4
Malaysia	1	0.5
Mexico	2	0.9
Netherlands	1	0.5
New Zealand	1	0.5
Norway	3	1.4
Poland	1	0.5
Portugal	3	1.4
Qatar	1	0.5
Serbia	1	0.5
Singapore	2	0.9
South Africa	1	0.5
Spain	6	2.8
Sweden	1	0.5
Switzerland	7	3.2
Taiwan	1	0.5
Thailand	1	0.5
Türkiye	4	1.8
Ukraine	1	0.5
United Arab Emirates	3	1.4
United Kingdom	11	5.1
United States of America	55	25.3

The survey link was disseminated via email by the European Association for Osseointegration (EAO) Office to experts selected and approved by the Scientific Task Force. Each organization had discretion over the expert selection process, which included members from leading international implant dentistry organizations, such as the Academy of Osseointegration (AO), the EAO, the International Team for Implantology (ITI), and the Osteology Foundation (OF).

The survey was initiated on October 3, 2024, and remained open for responses for 3 weeks until October 25, 2024. A reminder was sent on October 19, 2024. Participation in the survey was voluntary, with no incentives offered. The survey was conducted electronically through Microsoft FORMS (Redmond, WA, US). Informed consent was obtained from all participants before they could proceed with the survey. The first question of the survey explicitly asked participants for their consent to participate, outlining the purpose of the study, the voluntary nature of their involvement, and the confidentiality measures in place. If a participant declined to provide consent, they were automatically excluded. All data were collected, stored, and processed anonymously.

### 2.4 Agreement Definition

Even though there is no universally accepted method for defining consensus (Von Der Gracht 2012), this study applied the following thresholds for 7‐point Likert scale questions:

- *Strong consensus*: achieved when > 95% of the experts “somewhat agreed”, “agreed” or “strongly agreed” with the statement made, or alternatively when > 95% “somewhat disagreed”, “disagreed” and “strongly disagreed”;
- *Consensus*: defined when either agreement or disagreement was greater than 75% and no more than 95%;
- *No consensus*: defined when either agreement or disagreement was 75% or less.

For multiple‐choice questions allowing multiple responses, strong consensus for any single option was defined as selection by more than 95% of respondents, consensus as selection by more than 75% and no more than 95%, and no consensus as ≤ 75%.

### 2.5 Statistical Analysis

Each 7‐point Likert scale response was analyzed individually using descriptive statistics, with results presented as percentages. Additionally, the degree of agreement for each question was reported as the median score and interquartile range (IQR) (see Supplement), following the RAND guidelines (Khodyakov et al. 2023).

In the graphs, the percentage of agreement (%) was calculated by summing the responses “somewhat agree,” “agree,” and “strongly agree.” If the sum of “somewhat disagree”, “disagree” and “strongly disagree” was higher, that percentage was reported instead, with clarification provided in the figure footnotes. Summary graphs displaying the median and IQR were created following the RAND guidelines (Khodyakov et al. 2023) and presented in the supplement.

Data analysis was carried out using Microsoft Forms (Microsoft), STATA v18, and GraphPad Prism v10.

## 3 Results

Of the 217 experts who were contacted, 116 accessed the survey link, with all of them providing consent and successfully completing the questionnaire. This resulted in a response rate of 53.5%. To ensure confidentiality, no information regarding the countries of the respondents was collected, as including just one expert from specific countries could potentially compromise anonymity.

### 3.1 Professional Specialization and Working Environment

The vast majority of the experts reported holding either a single specialization degree (81%) or multiple degrees (14.7%). The most commonly represented specialties were periodontics (62.1%), followed by oral surgery (25%) and oral and maxillofacial surgery (17.2%). Prosthodontists comprised only 5.2% of the respondents, while a small percentage (4.3%) identified as general practitioners.

The majority of the experts (62.1%) reported working in one clinical setting, 31.9% in two, and 6% in three different working environments. “Private clinic” was the most commonly endorsed option (71.6%), followed by “University” (56.0%). “Public hospital” and “other” were selected overall by less than 20% of the experts.

### 3.2 Planning Phase

**FIGURE 2:**
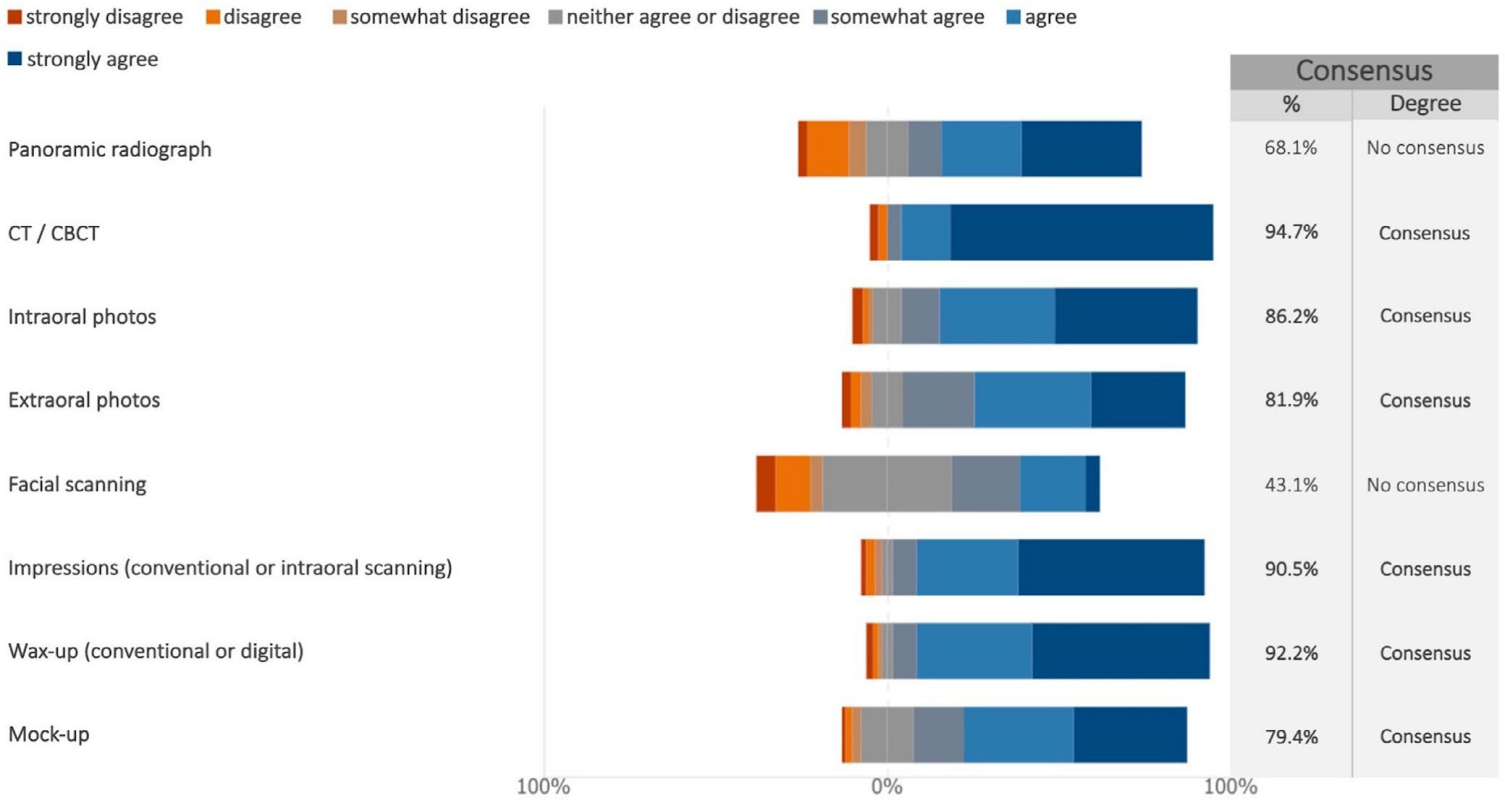
Which tool(s) should be routinely used for case study of maxillary implant‐supported full‐arch rehabilitation in combination with sinus lift or other bone grafting procedures?

The experts reached a consensus regarding several tools to be routinely used for case studies of maxillary implant‐supported full‐arch rehabilitation in combination with sinus lift or other bone grafting procedures (Figure 2; Figure S1). CT/CBCT emerged as a requisite tool, with the highest percentage of agreement among experts (94.7%) (median: 7; IQR: 7–7). Consensus was reached on the routine use of photographic documentation, impressions (conventional or digital), wax‐ups, and mock‐ups (Figure 2; Figure S1). Panoramic radiographs were deemed less relevant in the planning phase (68.1%) (median: 6; IQR: 4–7) together with facial scanning (43.1%) (median: 4; IQR: 4–5).

**FIGURE 3:**
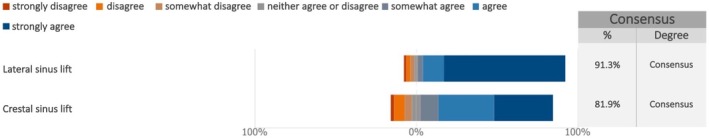
For sinus augmentation, do you consider it necessary to investigate by means of CT/CBCT the location and patency of the ostium, the presence of septae as well as the shape and route of the intrabony canal of the superior posterior alveolar artery?

The experts indicated that CT/CBCT was fundamental for a precise pre‐operative evaluation of the maxillary sinus anatomy (presence of septae, shape, alveolar artery), both in the case of lateral sinus lift (91.3%) (median: 7; IQR: 6.2–7) and crestal sinus lift (4%) (median: 6; IQR: 5–7) (Figure 3; Figure S2).

The experts were asked to specify the minimum subantral bone height (in mm) allowing for the performance of a lateral or a crestal sinus lift with simultaneous implant placement. For both approaches, a high variability was observed among the experts. For lateral sinus lift, the majority of experts indicated that 3–4 mm of subantral bone height is necessary for simultaneous implant insertion. However, a small percentage of respondents considered lower bone heights as viable options, with 5.2% advising 1 mm and 8.6% advising 2 mm. For the crestal approach, the most common recommendations for the minimum bone height were 5 mm (44.8%), followed by 4 mm (20.7%) and 6 mm (19.8%).

### 3.3 Guided Surgery

**FIGURE 4:**
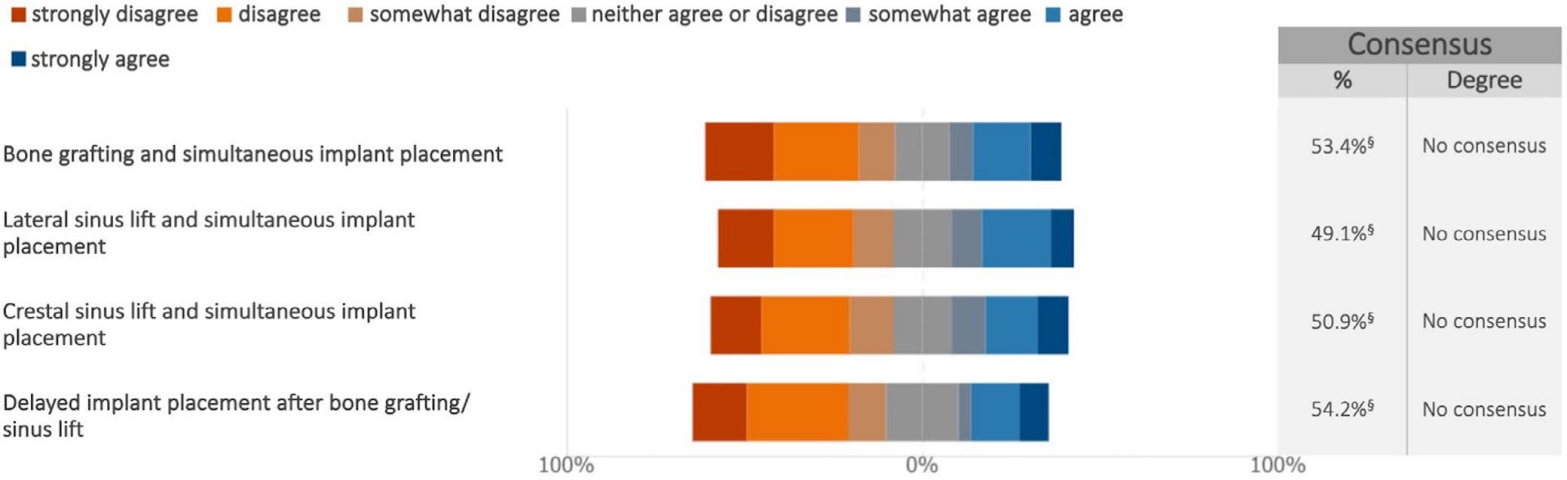
In case of multiple implant placements in fully edentulous maxilla, should freehand surgery be preferred over static/dynamic guided surgery? ^§^Sum of “somewhat disagree”, “disagree” and “strongly disagree” in %.

When considering multiple implant placement in fully edentulous maxilla, the experts tended to disagree on whether freehand surgery should be preferred over static or dynamic guided surgery, ultimately failing to reach a consensus (Figure 4; Figure S3).

### 3.4 Timing of Implant Placement and Loading

In cases of terminal dentition in the posterior maxilla with insufficient residual bone requiring sinus lift or bone grafting, the experts reached a consensus on the preferred implant placement protocol, with 76.7% favoring delayed implant placement over immediate or early placement.

In cases of primary bone augmentation or lateral sinus lift with delayed implant placement, the experts reported waiting times ranging from 2 to 10 months before performing the second surgery. Although 6 months (43.1%) and 4 months (19.%) were the most frequently preferred intervals, no consensus was reached on the optimal timing for implant placement.

**FIGURE 5:**
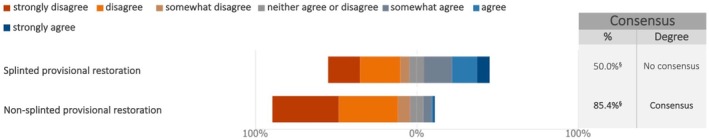
Is immediate loading protocol (within 1 week from implant placement) a secure treatment option in case of simultaneous sinus lift/bone grafting and good primary stability? ^§^Sum of “somewhat disagree”, “disagree” and “strongly disagree” in %.

The safety of immediate loading in cases of simultaneous sinus lift/bone grafting and good primary implant stability was also investigated (Figure 5; Figure S4). The experts showed clear disagreement regarding the use of non‐splinted provisional restorations (85.4%) (median: 2; IQR: 1–2). By contrast, no consensus was reached regarding the use of splinted provisional restorations, despite there being a prevailing tendency among the experts to discourage immediate loading even when splinted restorations were considered (median: 3.5; IQR: 2–5).

### 3.5 Biomaterials and Biologics (e.g., Blood Concentrates)

**FIGURE 6:**
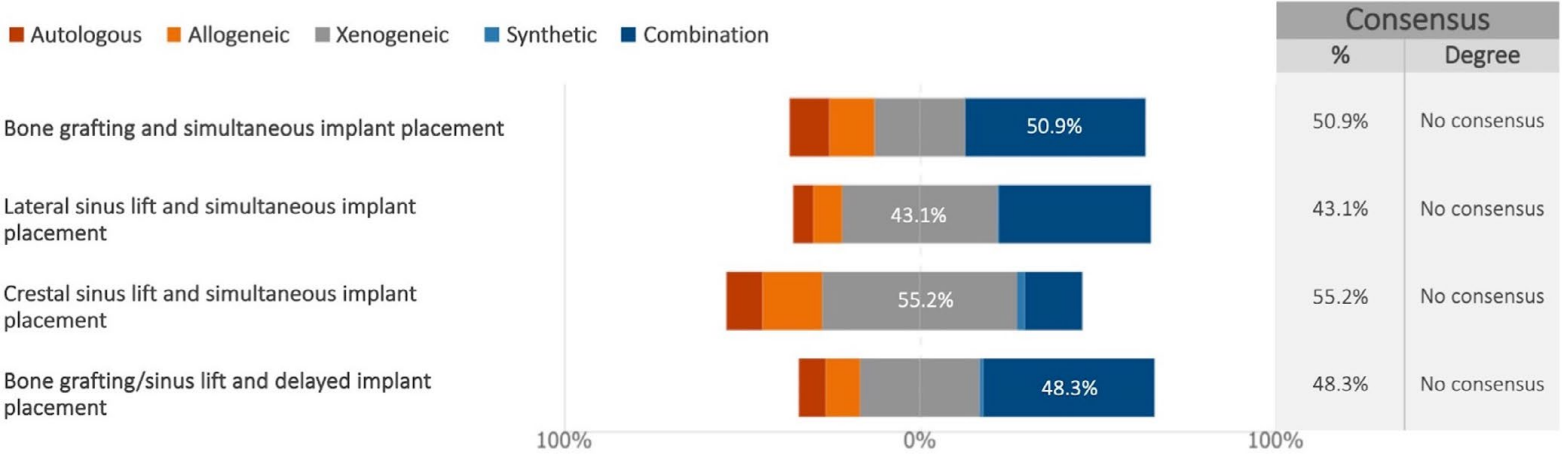
Which bone substitute material(s) do you preferably use among autologous, allogeneic, xenogeneic, synthetic ones, or a combination of various materials depending on the chosen surgical approach? % of the answers obtaining the highest response rate is reported.

The survey revealed a lack of overall consensus regarding the preferred bone substitute material, regardless of timing (primary or simultaneous augmentation). However, distinct patterns emerged when analyzing specific surgical procedures (Figure 6). For lateral and crestal sinus lift with simultaneous implant placement, xenogeneic materials were most frequently chosen by the experts. A combination of various biomaterials was favored for sinus lift and delayed implant placement, as well as for other bone grafting procedures.

**FIGURE 7:**
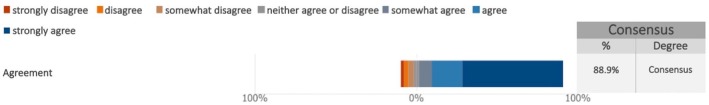
In case of GBR for vertical bone augmentation, should the membrane always be fixed (e.g., with pins, suture, etc.)?

**FIGURE 8:**
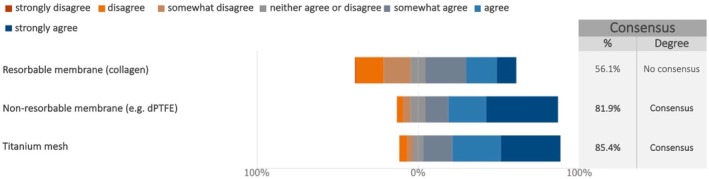
In your opinion, in case of GBR, does the membrane exposure have a detrimental influence on the outcome of bone regeneration?

Regarding guided bone regeneration (GBR) for vertical bone augmentation, the experts reached consensus that the membrane should always be fixed, typically with pins, sutures, or other fixation methods, to ensure proper placement and stability during the healing process (median: 7; IQR: 6–7) (Figure 7; Figure S5). In addition, the experts reached consensus on the negative impact of titanium meshes and non‐resorbable membranes on bone regeneration outcomes. Conversely, no consensus was reached regarding the impact of resorbable membrane exposure on bone regeneration outcomes (Figure 8; Figure S6).

**FIGURE 9:**
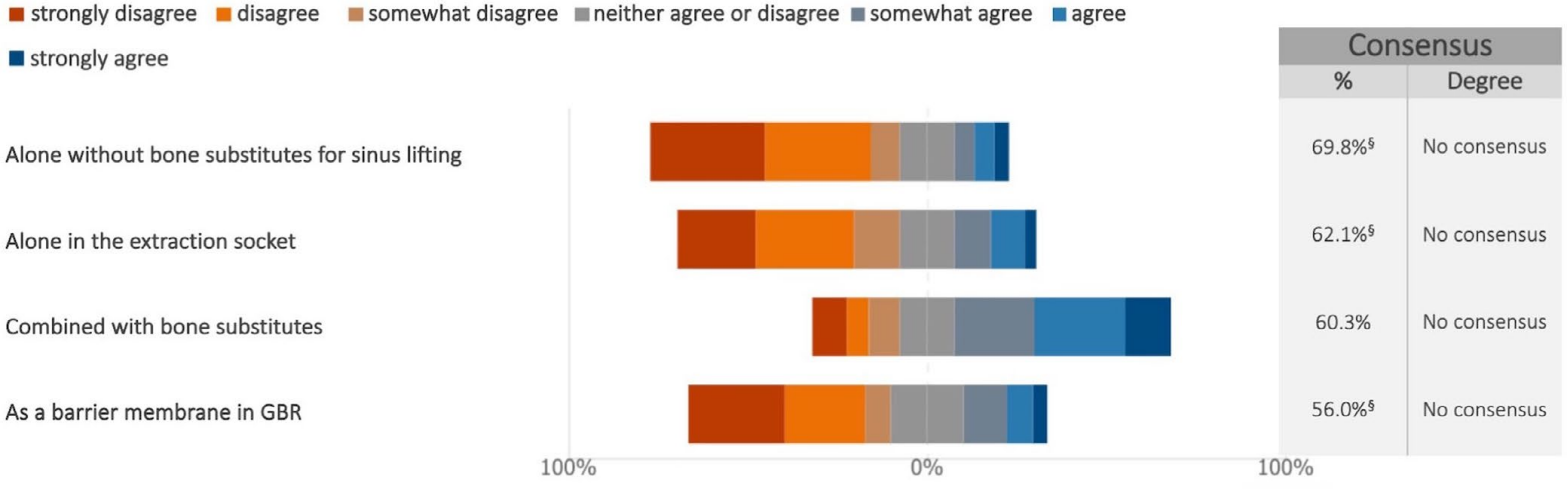
Do you consider biologics (e.g., blood concentrates) as appropriate treatment options for the following indications? ^§^Sum of “somewhat disagree”, “disagree” and “strongly disagree” in %.

The experts did not reach consensus on the use of biologics, such as blood concentrates, as appropriate treatment options for any of the proposed indications. Experts generally tended to disagree on the use of biologics as barrier membrane for GBR, as well as alone for socket preservation and sinus lift, suggesting reservations about their effectiveness in these contexts without additional treatments/biomaterials (Figure 9; Figure S7). However, some agreement was observed (60.3%) when biologics were combined with bone substitutes.

### 3.6 Provisional Restorations

When experts were asked about their preferred type of provisional restoration during the healing period in fully edentulous ridges, there was high variability in the responses. Approximately 20% indicated a preference for avoiding the use of a prosthesis, at least during the initial months of healing. Over 25% suggested the use of temporary implant‐supported restorations. Additionally, soft relined dentures or removable temporary prostheses without flanges were also proposed as suitable options.

### 3.7 Antibiotic Prescription in Relation to Implant Placement

Consensus was not reached regarding the routine prescription of antibiotics as prophylaxis during multiple implant placements for the rehabilitation of the fully edentulous maxilla. However, experts generally tended to prescribe antibiotics, with 68.1% indicating they always do so, and 26.7% prescribing them in certain circumstances, such as in medically compromised patients, lateral sinus lift, GBR, and bone blocks. Similar findings were recorded regarding the use of antibiotics following implant insertion, with 68.9% of experts claiming that they always prescribe antibiotic therapy after surgery as prophylaxis and 27.6% only in specific circumstances.

### 3.8 Maintenance

**FIGURE 10:**
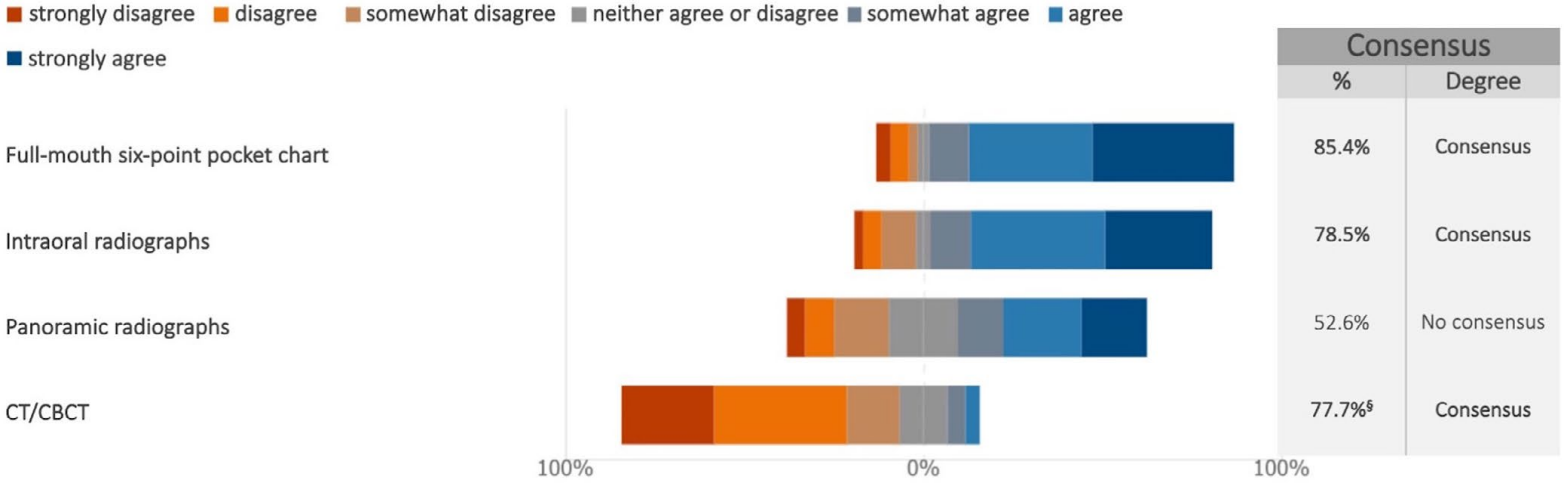
In absence of complications, are the following procedures justified at least once a year in the long‐term follow‐up? ^§^Sum of “somewhat disagree”, “disagree” and “strongly disagree” in %.

The experts reached consensus on the justification of performing full‐mouth pocket charts (85.4%) (median: 6; IQR: 5–7) and intraoral radiographs (78.5%) (median: 6; IQR: 5–7) at least once a year during long‐term follow‐up in the absence of complications, while the experts expressed disagreement concerning the routine use of CBCT (77.7%) (median: 2; IQR: 1–3) (Figure 10; Figure S8). No consensus was reached regarding the regular use of panoramic radiographs in the maintenance phase.

### 3.9 Future Trends

**FIGURE 11:**
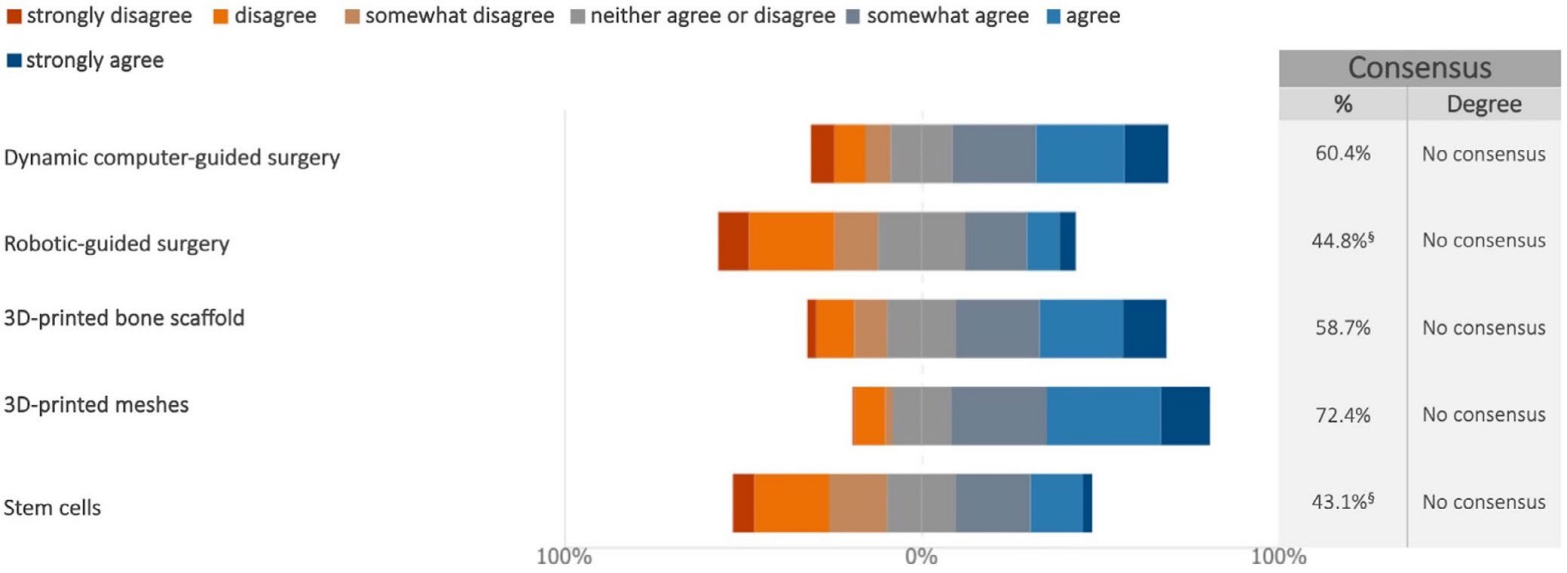
Do you think that the following materials/techniques will be commonly applied within the next 5 years for the rehabilitation of the fully edentulous maxilla with dental implants and regenerative techniques? ^§^Sum of “somewhat disagree”, “disagree” and “strongly disagree” in %.

No consensus was reached among the experts regarding the potential use of any of the listed emerging materials and techniques for rehabilitating the fully edentulous maxilla within the next five years. This includes dynamic and robotic guided surgery, 3D printed scaffolds and meshes, as well as stem cells (Figure 11; Figure S9). Interestingly, over 40% of the experts expressed disagreement regarding the widespread adoption of robotic surgery and stem cells for this indication within this time frame.

### 3.10 Schneiderian Membrane Perforation

**FIGURE 12:**
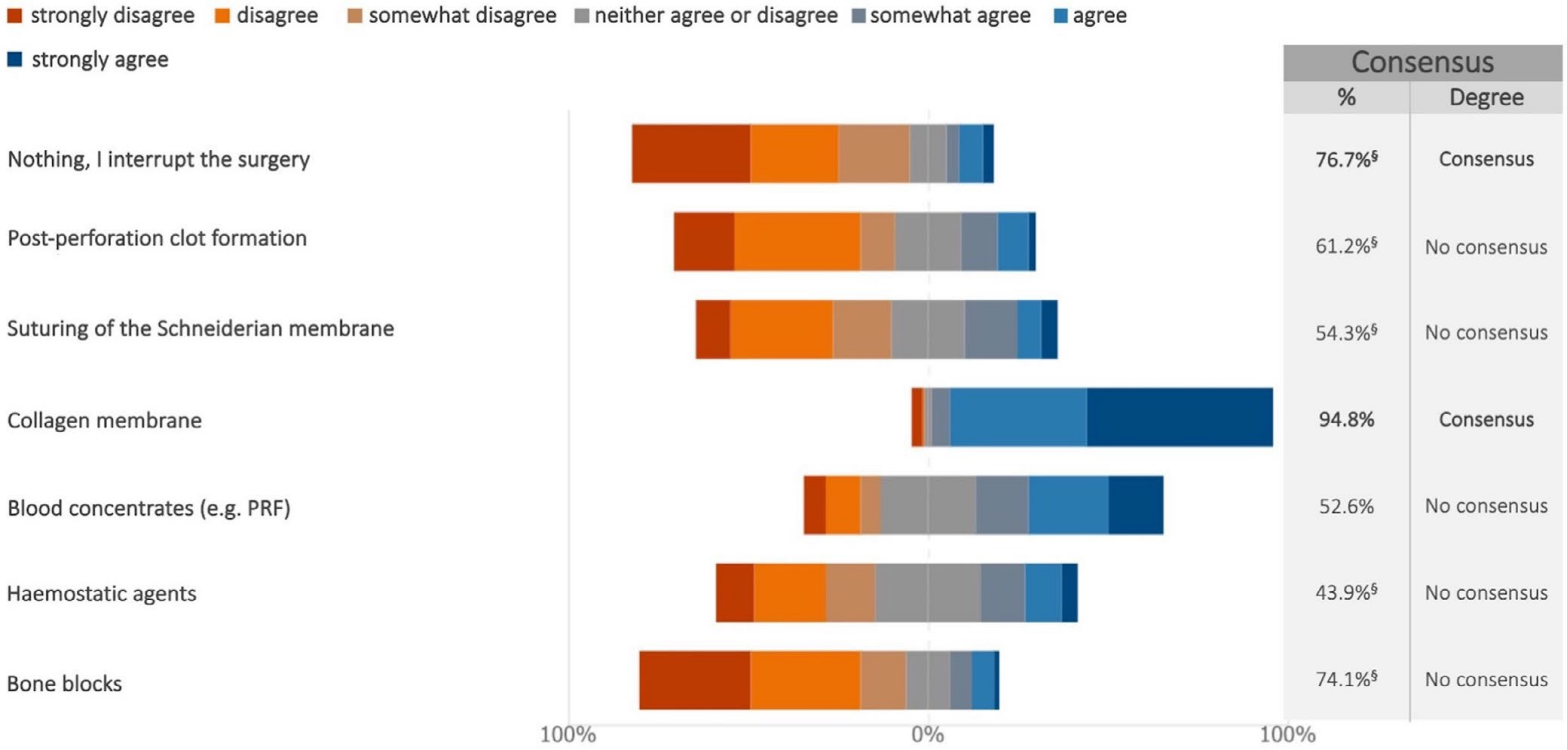
In case of perforation of the Schneiderian membrane during lateral sinus lift, to what extent do you consider the following options suitable? Perforation up to 5 mm. ^§^Sum of “somewhat disagree”, “disagree” and “strongly disagree” in %.

**FIGURE 13:**
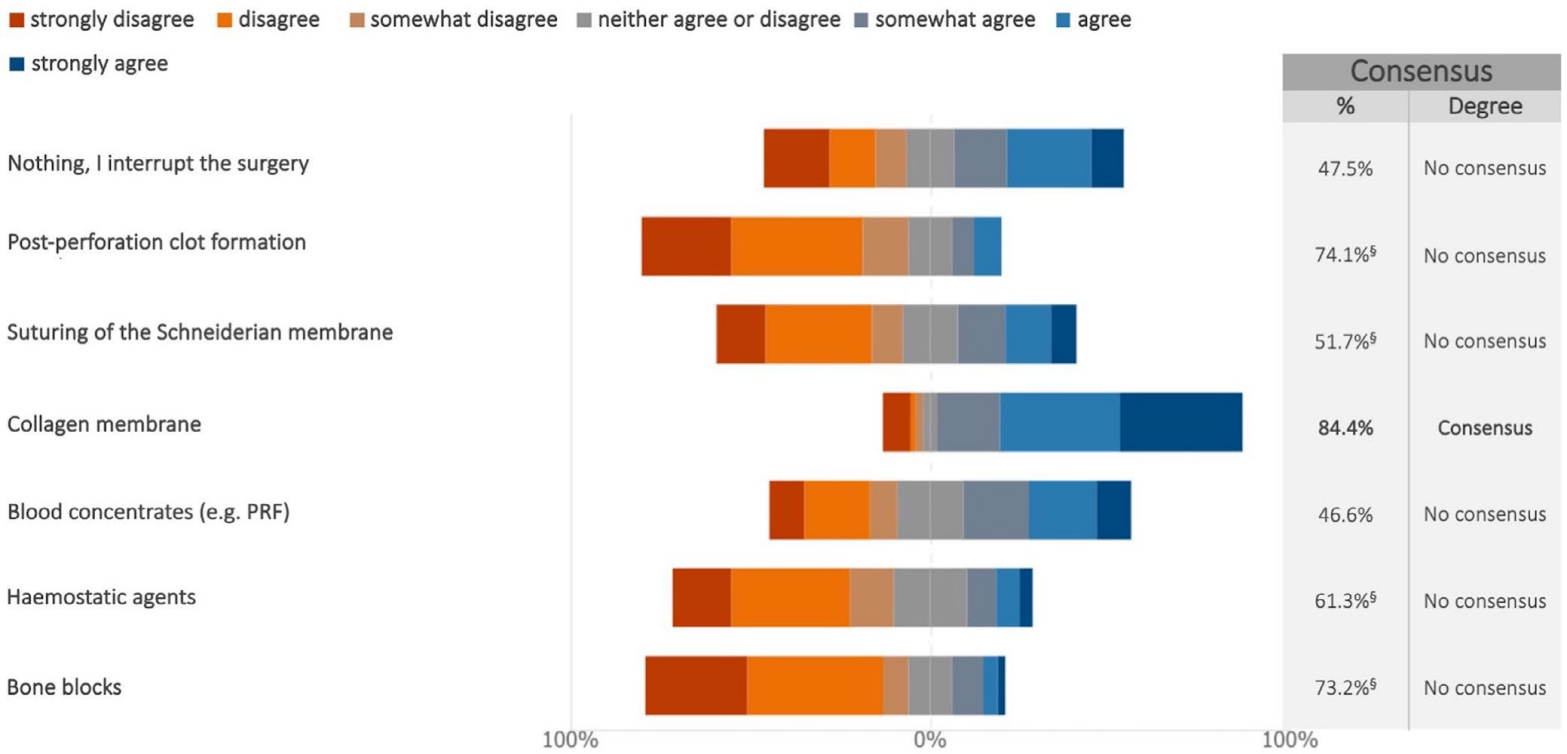
In case of perforation of the Schneiderian membrane during lateral sinus lift, to what extent do you consider the following options suitable? Perforation between 5 and 10 mm. ^§^Sum of “somewhat disagree”, “disagree” and “strongly disagree” in %.

**FIGURE 14:**
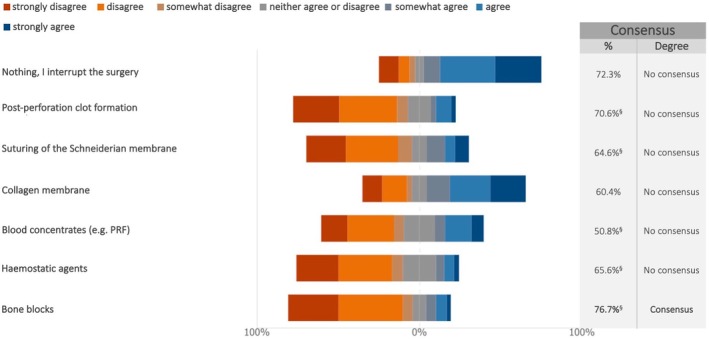
In case of perforation of the Schneiderian membrane during lateral sinus lift, to what extent do you consider the following options suitable? Perforation greater than 10 mm. ^§^Sum of “somewhat disagree”, “disagree” and “strongly disagree” in %.

In case of Schneiderian membrane perforation during lateral sinus lift, the experts reached a consensus on the suitability of the following procedures, which varied depending on the size of the perforation. For perforations up to 5 mm, experts disagreed on interrupting the surgery (76.7%) (median: 2; IQR: 1–3) and agreed on the usage of resorbable membranes (94.8%) (median: 7; IQR: 6–7) (Figure 12; Figure S10). For perforations between 5 and 10 mm, the experts supported the use of collagen membranes (84.4%) (median: 6; IQR: 5–7) (Figure 13; Figure S11), while for perforations greater than 10 mm, bone blocks were not considered an appropriate option (76.7%) (median: 2; IQR: 1–3) (Figure 14; Figure S12).

### 3.11 Soft Tissue Augmentation

In cases of insufficient keratinized tissue, consensus was reached regarding the timing of soft tissue augmentation to establish keratinized mucosa around dental implants. Indeed, 83.6% of experts stated that they generally perform the procedure either at the time of implant insertion or during the second‐stage surgery.

### 3.12 Implant Longevity

The experts agreed that the estimated longevity of implant treatment without complications under normal circumstances would exceed 10 years when implants are placed in native bone (87.1%) or in combination with lateral and crestal sinus lift (76.7% and 78.4%, respectively). While more than 10 years was the most commonly reported expected lifespan also for implants placed with GBR (65.5%) or bone blocks (59.5%), no consensus was reached in these cases.

### 3.13 Factors Affecting Patient Satisfaction

**FIGURE 15:**
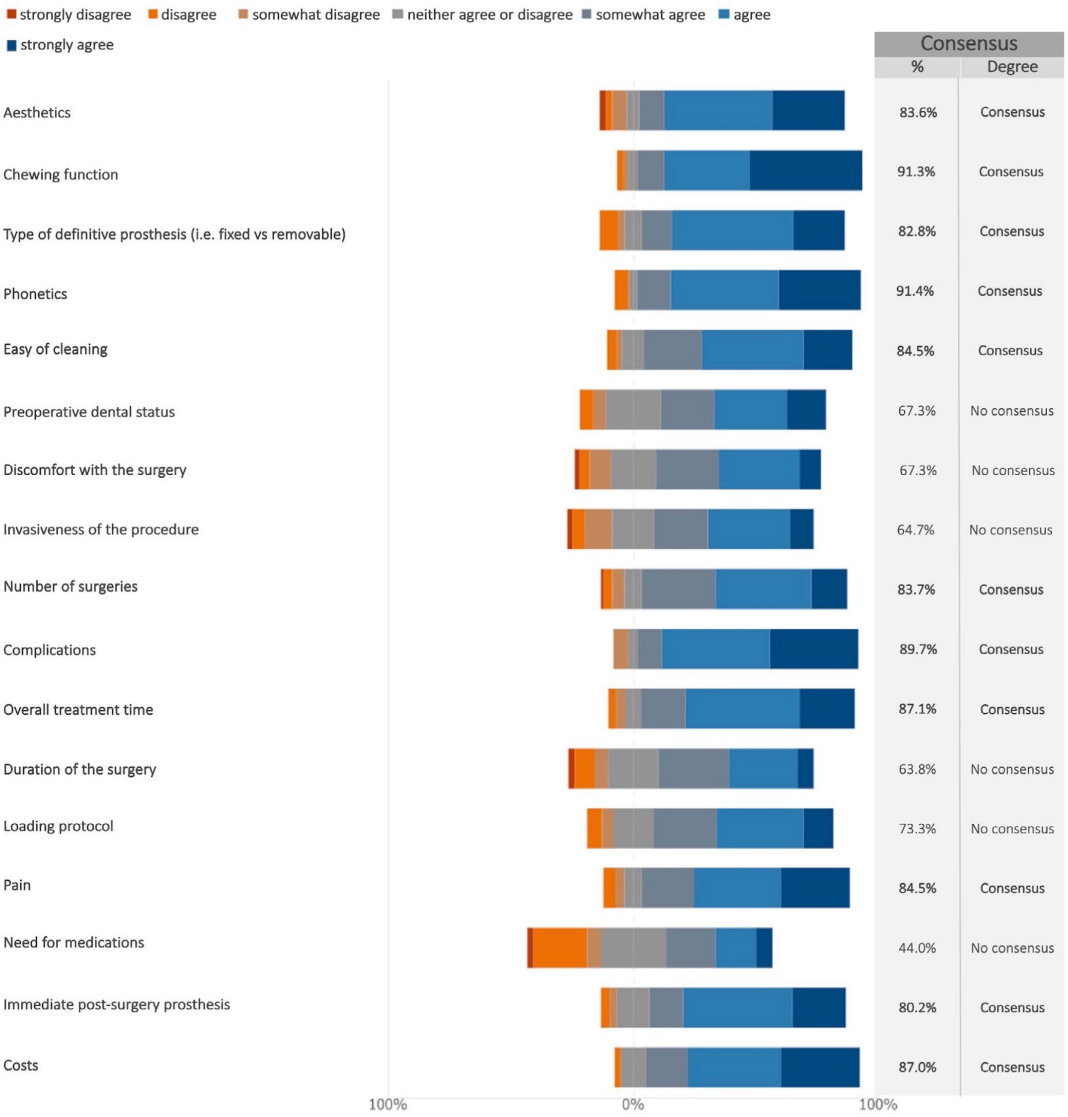
In case of maxillary full‐arch rehabilitation with dental implants requiring sinus grafting/alveolar ridge augmentation, do the following factors affect the overall patient satisfaction in the short term?

In the case of maxillary full‐arch rehabilitation with dental implants requiring sinus grafting or alveolar ridge augmentation, consensus was reached on several factors influencing overall patient satisfaction in the short term (Figure 15; Figure S13). Notably, agreement exceeding 90% was reached on the importance of chewing function and phonetics as key determinants of patient satisfaction.

### 3.14 Factors Influencing Selection of Treatment Procedures

**FIGURE 16:**
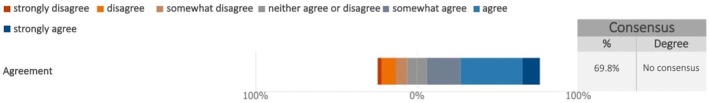
How important is the difficulty of the procedure when you choose one?

The selection of the treatment option was found to be influenced by the available evidence (27.6%), often combined with the perceived ease of the procedure (71.6%). The ease of treatment alone was considered adequate for making a decision just by one expert. No consensus was reached regarding the significance of technical difficulty in treatment selection, with 69.8% agreement on the matter (median: 5; IQR: 4–6) (Figure 16; Figure S14).

### 3.15 Fundamental Outcomes to Be Included in Future Studies

**FIGURE 17:**
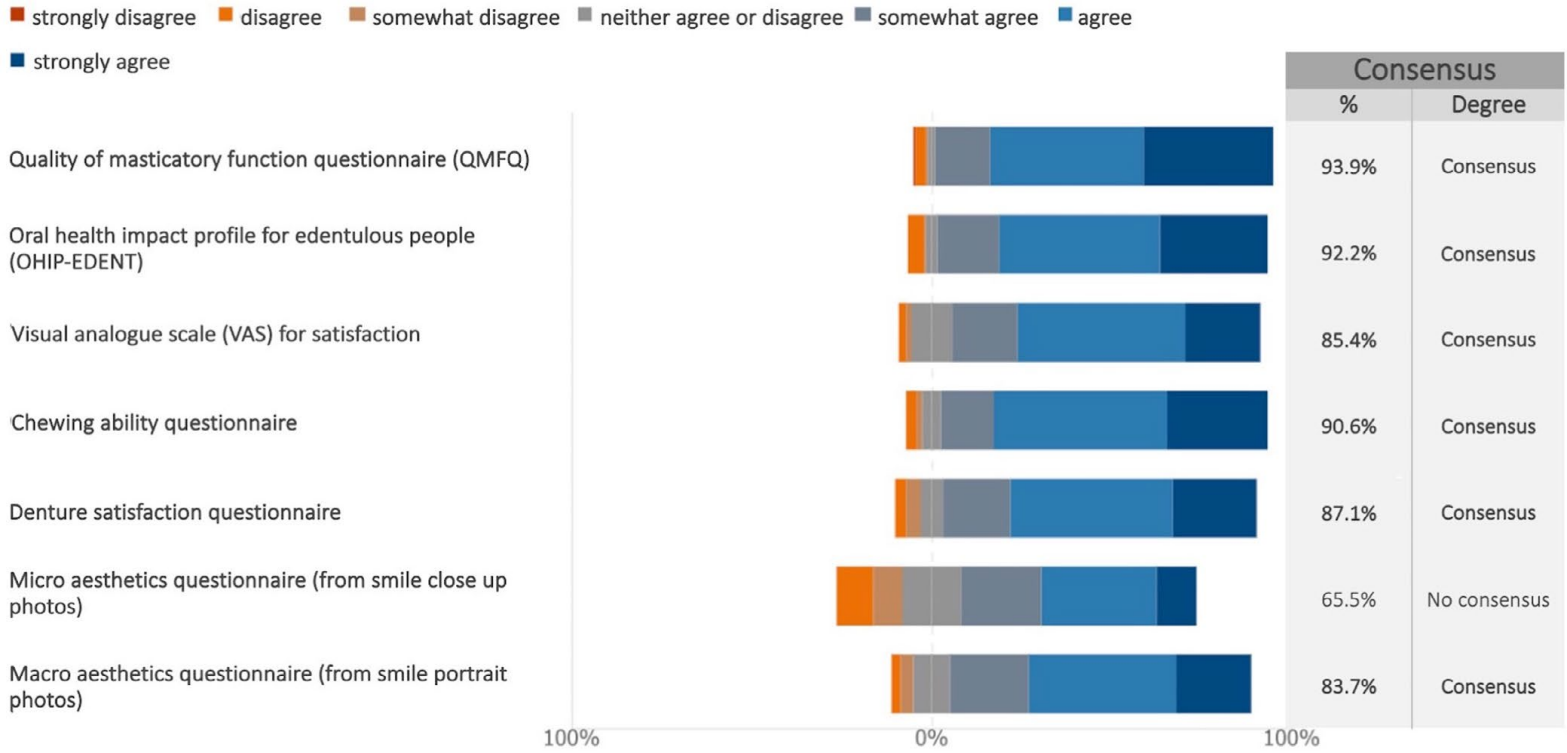
In future studies on maxillary full‐arch rehabilitation with dental implants, do you consider relevant the following patient‐reported outcome measures (PROMs)?

In future studies on maxillary full‐arch rehabilitation with dental implants, all the proposed patient‐reported outcome measures (PROMs) were considered relevant, with the exception of the micro aesthetics questionnaire (from smile close‐up photos), for which no consensus was reached (Figure 17; Figure S15).

**FIGURE 18:**
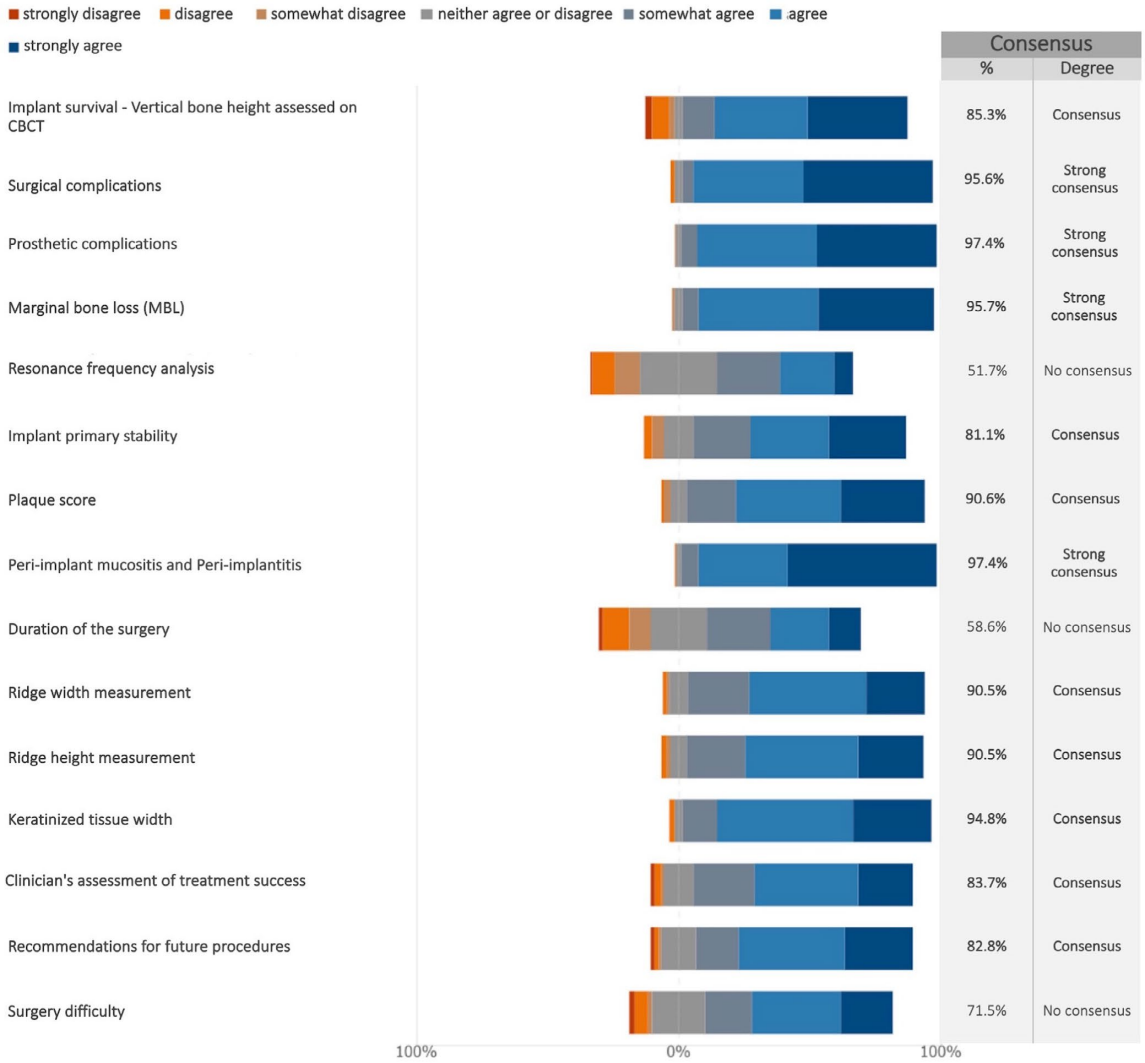
In future studies on maxillary full‐arch rehabilitation with dental implants, do you consider relevant the following clinician‐reported outcomes ClinROs?

A variety of clinician‐reported outcomes ClinROs were deemed relevant to include in future studies. In particular, strong consensus was reached on the significance of assessing surgical, prosthetic, and biological complications, as well as marginal bone loss (Figure 18; Figure S16).

## 4 Discussion

The aim of the present study was to gather expert opinions on sinus lift and alveolar bone augmentation techniques for rehabilitating the edentulous maxilla using a single‐round survey. The findings were then compared with existing evidence to inform a consensus development process and identify areas where additional systematic reviews or research are needed. The main results of the survey are as follows:

### 4.1 Planning Phase

CT/CBCT scans were deemed essential for maxillary implant planning (94.7%) and anatomical assessment, while panoramic radiographs and facial scanning were considered secondary.

The use of diagnostic imaging techniques such as CT and CBCT in the planning phase is supported also by other authors (Katsoulis et al. 2009; Valentini and Artzi 2023). They provide comprehensive information on anatomical and pathological features, including thickness of the lateral bony wall of the maxillary antrum, presence and location of Underwood's septa, Schneiderian membrane thickness, and presence of sinus pathologies (Valentini and Artzi 2023; Varela‐Centelles et al. 2015).

The experts agreed that intraoral photographs, impressions, wax‐ups, and mock‐ups are valuable supplementary tools for treatment planning (Papaspyridakos et al. 2022). Incorporating data from intraoral or facial scanners during the planning phase might provide additional benefits (Hou et al. 2022).

Considerable variability was observed among the experts concerning the minimum subantral bone height required for sinus lift procedures, reflecting the lack of standardized criteria, with clinical decisions often relying on practitioner experience and preference. While some evidence suggests that the crestal approach is preferable when residual bone height exceeds 6 mm and the lateral approach is indicated below 4 mm, cases falling between these values often depend on specific anatomical and procedural factors, including the number of planned implants and the width of the sinus (Lyu et al. 2023; Testori et al. 2024). Interestingly, our survey found that for lateral sinus lift, the majority of experts indicated that 3–4 mm of subantral bone height is necessary for simultaneous implant insertion. For the crestal approach, the most common recommendations for minimum bone height were 5 mm (44.8%), followed by 4 mm (20.7%), and 6 mm (19.8%).

### 4.2 Guided Surgery

There was no consensus on whether freehand surgery should be preferred over static or dynamic guided techniques for multiple implant placement. This may be due, in part, to the limited number of studies that have directly compared freehand surgery with static or dynamic guidance. A recent systematic review (Aghaloo et al. 2023) emphasized the need for such comparative studies to evaluate true clinical efficacy. Nearly half of the respondents expressed opposition to the freehand approach, regardless of the specific procedure, such as bone grafting with simultaneous implant placement or sinus lift procedures with or without implants.

While freehand placement is often considered the minimum standard of care, its success is highly dependent on the clinician's experience and skill. The growing availability and adoption of guided surgical techniques may also explain the experts' answers. According to a systematic review and meta‐analysis (Gargallo‐Albiol et al. 2020), static fully guided implant surgery offers the highest accuracy in transferring the presurgical plan to the patient, followed by static half‐guided methods, with freehand surgery being the least accurate. Another more recent systematic review with meta‐analyses found that guided techniques do not negatively impact implant surgery outcomes. Moreover, a recent non‐randomized prospective study (Jaemsuwan et al. 2023) demonstrated that both static and dynamic CAIS significantly outperformed the freehand approach in terms of accuracy in fully edentulous patients.

Robotic‐assisted dental implant surgery demonstrated superior accuracy in coronal, apical, and angular deviations when compared to freehand, static, and dynamic techniques (Sankar et al. 2025). However, the authors warned that the limited number of clinical studies and potential funding biases could affect the reliability of these findings, making cautious interpretation necessary.

### 4.3 Timing of Implant Placement and Loading

When asked about the preferred protocol for implant placement with terminal dentition in the posterior maxilla and inadequate residual bone requiring sinus lift or bone grafting, the majority (76.7%) favored delayed implant placement over immediate or early placement. Reported waiting times varied between 2 and 10 months, with no consensus on the ideal duration. A recent consensus focused on the rehabilitation of the posterior atrophic maxilla concluded that sinus floor augmentation with simultaneous implant placement must account for bone height, width, quality, and clinician experience (Testori et al. 2024). Furthermore, no minimum crestal height could be defined, as multiple factors influence the risk of implant displacement into the sinus (Seigneur et al. 2023; Testori et al. 2024).

Experts largely opposed immediate loading, especially for non‐splinted restorations (85.4%). These findings are not consistent with previous systematic reviews indicating that immediate implant loading with a fixed prosthesis in the edentulous maxilla seems to be a reliable treatment alternative (Jiang et al. 2021) with similar survival rates to delayed loading (Papaspyridakos et al. 2014). However, these systematic reviews did not specifically address implant loading protocols in conjunction with sinus lift procedures or other bone grafting techniques. Moreover, they fail to distinguish between immediate implant placement with immediate loading and immediate loading following delayed implant placement in previously regenerated bone.

### 4.4 Biomaterials and Biologics

No consensus emerged on the preferred bone substitute material, though xenogeneic grafts were favored for sinus lifts. The review and meta‐analysis by Starch‐Jensen and colleagues found that synthetic bone substitutes are as effective as other grafting materials (such as autografts, allografts, and xenografts) for maxillary sinus floor augmentation in terms of clinical outcomes, such as implant survival and bone regeneration (Starch‐Jensen et al. 2020). The 5‐year implant survival after sinus lift procedure with autogenous bone graft was 97%, compared to 95% for Bio‐Oss (Starch‐Jensen, Aludden, et al. 2018); additionally, the reduction in vertical height of the augmented sinus was similar for both treatment modalities (Starch‐Jensen, Mordenfeld, et al. 2018). Bone substitutes, in oral bone augmentation procedures in general, and in sinus augmentation in particular, have been shown to be an adequate grafting source (Valentini and Artzi 2023). Another review indicated that bone substitutes might be as effective as autografts and serve as an alternative with less morbidity (Raghoebar et al. 2019). All the above cited is in line with the experts' preferences to use xenografts, since the literature data indicate their success in these procedures, associated with decreased morbidity.

Experts agreed that membrane fixation plays an important role in GBR for vertical bone augmentation. This aligns with the widely accepted understanding that fixation enhances membrane stability, preventing its collapse (Wang and Boyapati 2006). This stability enables the formation of an adequate blood clot, creating favorable conditions for bone regeneration (Hammerle et al. 1995; Wang and Boyapati 2006). Biologically, fixation may also increase the expression of osteogenic factors as demonstrated in a preclinical study (An et al. 2022), further supporting GBR.

Although a recent systematic review by (Wessing et al. 2018) reported an advantage of membrane fixation in terms of bone gain, there is still limited systematic evidence directly comparing fixation versus no fixation. Additionally, while recent reviews have shown that vertical bone gains of approximately 4.2 mm (Sabri et al. 2024) can be achieved using GBR and 3.3 mm with titanium meshes (Urban et al. 2019) they did not consistently differentiate between studies that employed membrane fixation and those that did not—leaving an important gap in the current evidence base.

While experts consistently agreed on the negative effects of membrane exposure for titanium and non‐resorbable membranes, opinions were divided regarding the impact of exposures on resorbable membranes. This potential advantage of resorbable membranes may lie in their ability to support spontaneous healing after exposure (Jin et al. 2023) along with simpler clinical management in these situations.

Previous systematic reviews (Garcia et al. 2018; Sanz‐Sánchez et al. 2015) and narrative reviews (Donos et al. 2023) have reported that membrane exposure negatively impacts bone regeneration outcomes, suggesting the importance of achieving primary intention healing in GBR procedures (Garcia et al. 2018). While a recent RCT found that non‐resorbable membranes offer superior dimensional stability compared to resorbable membranes (Strauss et al. 2025) these differences may have limited long‐term clinical significance (Naenni et al. 2021). Regarding titanium meshes, recent systematic reviews with meta‐analyses have reported exposure rates ranging from 10.8% (Sabri et al. 2024) to 19.9% for conventional titanium meshes and 15.2% for 3D‐customized titanium meshes (Gu et al. 2022).

The use of biologics (e.g., growth factors, blood derivatives) has gained popularity in modern implant dentistry, driven by the belief that these agents can improve regenerative outcomes (Avila‐Ortiz et al. 2022). However, the experts did not support any of the proposed indications—such as their use alone without bone substitutes, for sinus lift, alveolar ridge preservation, or as a barrier for GBR—except when combined with bone substitutes (supported by 60.3% of the experts). These findings align with existing systematic (Arora et al. 2025; Miron et al. 2017; Stähli et al. 2018; Strauss et al. 2018), narrative reviews (Blanco et al. 2025; Galarraga‐Vinueza et al. 2024; Quirynen et al. 2025; Valentini et al. 2025) which highlight the limited evidence available, restricting the broad application of biologics and raising concerns about their cost efficacy.

### 4.5 Provisional Restorations

Preferences varied, with 20% favoring no prosthesis during early healing, over 25% opting for temporary implant‐supported restorations, and others recommending removable prostheses. Literature lacks data on guidelines for provisional restorations in the healing phase, and usually it is up to the clinician's preference and choice. A previous survey in the United States, which was responded by 47 prosthodontists, revealed that 67% preferred to deliver a complete‐arch fixed provisional prosthesis at the time of surgery (Schoenbaum et al. 2020). Following the augmentation process of the edentulous maxilla, there is a clinical preference to avoid mobile restorations due to their possible negative effects on the augmented sites, which has been proposed by 20% of experts in this survey as well.

However, in cases in which immediate implant placement is performed, immediate provisionalization can be advised. Additionally, there is a possibility of the use of provisional implants to stabilize a reconstruction. A 10‐year retrospective analysis investigated full‐arch rehabilitation using immediate dental implant placement and continuous functional loading with full‐fixed dental prostheses (FFDPs) (Diez‐Fraile et al. 2024). The study involved 56 patients who received temporary implants at maxillary augmentation sites. The findings indicated a high survival rate of provisional implants (97.9%) and definitive implants (92.2%) over the decade, with moderate marginal bone loss observed. This underscores the viability of immediate provisionalization in long‐term rehabilitation strategies.

### 4.6 Antibiotic Prescription in Relation to Implant Placement

There is a scarce data on the guidelines for antibiotic prescriptions, and this should be incorporated in future research. A clinical consensus from more than 10 years ago about sinus lift suggests administering antibiotics both preoperatively and postoperatively to reduce infection risks (Testori et al. 2012).

While no consensus was reached, most experts routinely opt for antibiotic prescriptions (68.1%) or did so in specific cases (26.7%), including medically compromised patients, sinus lifts, and GBR. Similar trends were observed postoperatively, although no clear, universally accepted guidelines are available for antibiotic administration in healthy and medically compromised individuals.

### 4.7 Maintenance

The maintenance phase is critical to the long‐term success of implant‐supported full‐arch rehabilitation, particularly in complex maxillary reconstructions involving sinus lifts and bone augmentation. The experts' consensus on conducting full‐mouth pocket charts at least once a year (85.4%) highlights the importance of peri‐implant soft tissue monitoring in detecting early signs of inflammation, mucositis, or peri‐implantitis. This is in line with current literature endorsing peri‐implant probing as a non‐invasive and valuable diagnostic tool (Berglundh et al. 2018; Lang and Berglundh 2011), and recent evidence further supports its safety when performed correctly (Monje and Salvi 2024). Although the European Federation of Periodontology S3 guideline does not currently recommend a specific time interval for supportive peri‐implant care, intervals of 3, 6 or 12 months should be individualized based on patient‐specific risk profiles, as regular supportive care plays a crucial role in reducing the risk of peri‐implant diseases in patients with healthy peri‐implant tissues (Herrera et al. 2023).

The consensus on annual intraoral radiographs (78.5%) reflects the experts' preference for localized imaging as the gold standard for assessing marginal bone levels and detecting early bone loss. This aligns with international guidelines, which recommend periodic periapical imaging for baseline and longitudinal assessments (Jepsen et al. 2015; Renvert et al. 2018).

In contrast, a considerable number of experts disagreed with the routine use of CBCT (77.7%) during maintenance, recommending its use be reserved for specific clinical indications due to concerns about radiation exposure and cost (Bornstein et al. 2014; Sahrmann et al. 2024). The lack of consensus on panoramic radiographs likely reflects their limited diagnostic sensitivity compared to targeted intraoral techniques.

Altogether, these findings support a conservative, patient‐centered approach to maintenance, emphasizing low‐risk, high‐yield diagnostics, with future research needed to refine follow‐up intervals and imaging strategies tailored to individual risk profiles and case complexity.

### 4.8 Future Trends

No consensus was reached on the adoption within the next 5 years of emerging technologies such as robotic surgery, dynamic navigation, 3D‐printed scaffolds, and stem cell therapies for the rehabilitation of the fully edentulous maxilla. Notably, over 40% of experts expressed skepticism about the widespread adoption of robotic surgery and stem cells within this timeframe, reflecting a cautious position toward innovations still in early clinical stages.

While innovations like robotic and dynamic navigation offer improved surgical accuracy, their widespread implementation is hindered by cost, training requirements, and a lack of long‐term outcome data (Bahrami et al. 2024; Jain et al. 2023). Similarly, although 3D‐printed scaffolds and meshes show potential for customized bone regeneration, their widespread use is limited by regulatory, mechanical, and material challenges (Davidopoulou et al. 2024; Vaquette et al. 2021). Stem cell therapy also holds promise for regenerative applications but remains largely experimental due to ethical, logistical, and regulatory constraints, and clinical translation is still in its early stages (Ivanovski et al. 2024; Zhao et al. 2023).

Overall, these findings indicate that clinicians continue to favor predictability and clinical feasibility over rapid integration of novel technologies. Future research, including well‐designed clinical trials, will be crucial to define the role of these innovations in routine care, particularly for complex maxillary reconstructions.

### 4.9 Schneiderian Membrane Perforation

The management of Schneiderian membrane perforations during lateral sinus lift procedures is essential for maintaining graft stability and optimizing implant success. The experts reached a consensus that small perforations (≤ 5 mm) can generally be managed without interrupting the procedure (76.7%), most commonly using resorbable membranes (94.8%). Narrative reviews (Molina et al. 2022; Testori et al. 2023) suggest that small perforations (≤ 5 mm) may not require repair, as the membrane often folds over itself when elevated from the sinus floor and surrounding bony walls. Nevertheless, the use of a collagen membrane or platelet‐rich fibrin to protect a perforated, elevated membrane is sometimes recommended (Testori et al. 2023).

For moderate perforations (5–10 mm), the preferred management strategy involves the use of collagen membranes (84.4%), due to their biocompatibility and ease of handling. These findings are consistent with systematic (Díaz‐Olivares et al. 2021) and narrative reviews (Molina et al. 2022; Testori et al. 2023) which indicate that for perforations measuring between 5 and 10 mm, the most commonly recommended treatment involves the use of a slowly resorbable collagen membrane. These membranes can favor tissue regeneration while promoting closure of the communication.

In contrast, for larger perforations (> 10 mm), the experts rejected the use of bone blocks (76.7%), citing concerns about increased surgical complexity and reduced predictability. In such scenarios, the literature supports a more conservative approach, such as delaying the augmentation or adopting staged sinus lift procedures. Graftless techniques in these cases may offer limited predictability regarding bone gain and long‐term stability (Lie et al. 2022; Pjetursson and Lang 2014).

Furthermore, recent insights highlight the importance of reducing invasiveness and proactively managing complications in sinus augmentation via the lateral window approach, emphasizing the refinement of surgical techniques to prevent membrane damage and improve overall clinical outcomes (Valentini and Artzi 2023). Ultimately, decision‐making should be guided by the size of the perforation, the clinician's surgical experience, and the availability of suitable materials, with an emphasis on minimally invasive yet effective techniques.

### 4.10 Soft Tissue Augmentation

Soft tissue management is an essential component of implant therapy, particularly in cases of insufficient keratinized mucosa, where it contributes to the preservation of peri‐implant health and long‐term clinical success. In this study, a clear consensus emerged among the experts, with 83.6% indicating that soft tissue augmentation is typically performed either at the time of implant placement or during second‐stage surgery to establish sufficient keratinized mucosa. This approach reflects the growing emphasis on early intervention to establish a stable soft tissue seal and minimize the risk of biological complications (Thoma et al. 2022).

The role of keratinized mucosa width in peri‐implant health remains a subject of ongoing debate in the literature. Several studies have suggested that an adequate width of keratinized tissue contributes to improved plaque control, reduced mucosal inflammation, and greater patient comfort (Thoma, Naenni, et al. 2018; Wennström and Derks 2012). A recent systematic review (Stefanini et al. 2023) demonstrated that augmentation techniques, such as free gingival grafts and connective tissue grafts, can effectively maintain healthy peri‐implant tissue over the medium and long term. Similarly, a network meta‐analysis (Tavelli et al. 2021) found that modifying the soft tissue phenotype—by increasing mucosal thickness and keratinized tissue—significantly improves peri‐implant health, reduces peri‐implant disease incidence, and enhances aesthetic outcomes.

In contrast, the EFP S3‐level clinical practice guideline on the “Prevention and Treatment of Peri‐Implant Diseases” concluded that the impact of soft tissue augmentation (“prophylactic” soft tissue augmentations) on peri‐implant health remains uncertain (Herrera et al. 2023). This ambiguity is echoed in recent reviews with conflicting results. For instance, a systematic review (Ramanauskaite et al. 2022) reported that a keratinized mucosa width of less than 2 mm is associated with increased soft tissue recession, marginal bone loss, plaque accumulation, and peri‐implantitis. However, another systematic review with meta‐analysis (Ravidà et al. 2022) found that having ≥ 2 mm of keratinized mucosa had minimal influence on peri‐implantitis development. Similarly, a 10‐year prospective study (Mancini et al. 2024) indicated that although the absence of buccal keratinized mucosa correlates with elevated peri‐implant parameters, such as bleeding on probing, the strength of this association appears weak, consistent with findings from a recent umbrella systematic review and meta‐analysis (Sabri et al. 2025).

### 4.11 Implant Longevity

Implants placed in native bone were expected to last over 10 years by 87.1% of respondents, with similarly high expectations for implants placed after sinus lifts (76.7%). These views are consistent with a recent systematic review and meta‐analysis, which reported a 10‐year implant survival rate of 96.4%, increasing even further for full‐arch prostheses (Howe et al. 2019). This is also in line with the largest cohort study to date, which showed an implant loss rate of 7.6% after 9 years of function. Furthermore, a secondary analysis of the same cohort found that edentulous patients were at a significantly higher risk of developing complications (hazard ratio: 3.2), consistent with previous studies that reported a hazard ratio of 2.4 for chipping and or loss of retention when comparing full‐arch reconstructions to single‐unit restorations (Karlsson et al. 2018).

No consensus was reached on longevity following GBR or bone block procedures. This uncertainty may be due to the wide variability in the results of these techniques in the edentulous maxilla, as well as the absence of recent systematic reviews reporting long‐term implant survival data (> 10 years) in grafted sites with. Although an earlier systematic review reported similar survival rates between grafted (GBR or bone blocks) and non‐grafted sites (Aghaloo et al. 2016), it primarily included retrospective studies, which may either underestimate or overestimate the true survival rates.

### 4.12 Factors Affecting Patient Satisfaction

Chewing function and phonetics were identified as key determinants of short‐term patient satisfaction, with over 90% agreement. Since edentulism negatively affects nutrition, mental health, and overall quality of life, the importance of effective rehabilitation is well recognized (Hunter et al. 2024). In a previous study (Linn et al. 2024), changes in oral health‐related quality of life (OHRQoL) were evaluated following different full‐arch prosthetic treatments in elderly edentulous patients. The findings suggest that implant‐supported prostheses significantly improve OHRQoL compared to conventional dentures. This highlights the substantial benefits of implant‐supported rehabilitations in terms of patient satisfaction and quality of life, particularly with regard to functional outcomes such as chewing efficiency and phonetics.

### 4.13 Factors Influencing Selection of Treatment Procedures

Decision‐making in treatment selection is increasingly influenced by a combination of available evidence, technological advancements, patient‐centered considerations as well as the clinician's expertise, clinical skills, personal values, and personal preferences (Tonetti et al. 2019) (Cosyn et al. 2023; Thoma et al. 2024). In the present study, decision‐making was influenced by a combination of available evidence and perceived ease of the procedure. However, no consensus was reached regarding the impact of technical difficulty.

Sanz et al. (2019) forecasted trends in implant dentistry, emphasizing a shift toward less invasive procedures and digital workflows, alongside the growing importance of patient‐centered outcomes in treatment planning. A review by Messias et al. (2023) further emphasized the variability in outcome assessments in full‐arch implant prosthetics and called for standardized criteria to support clearer treatment selection.

### 4.14 Fundamental Outcomes to Be Included in Future Studies

A PROM is a questionnaire designed to assess a patient‐reported outcome (PRO), capturing aspects of the patient's treatment experience that cannot be obtained through other means (Komagamine et al. 2023). All but one of the proposed PROMs reached consensus. These results align with earlier systematic reviews that highlight the increasing focus on PROMs in implant dentistry (De Bruyn et al. 2015; Feine et al. 2018; Yao et al. 2018). It has been suggested that when patients complete PROMs and this information is shared with clinicians, it can enhance communication, leading to better treatment processes and outcomes (Nelson et al. 2015; Sutherland and Till 1993). PROMs may also help patients articulate their values and concerns more clearly, making them easier for clinicians to understand and encouraging more active involvement of the patients in their care (Feldman‐Stewart and Brundage 2009; Santana and Feeny 2014).

Most ClinROs achieved either consensus or strong consensus. However, many studies focused on the edentulous maxilla fail to assess all outcomes relevant from a clinician's perspective. This lack of consistency limits the ability to compare results across studies. A practical solution is the adoption of a Core Outcome Set (COS), which defines a standardized set of essential outcomes to be routinely measured and reported (Clarke 2007). In implant dentistry, recent efforts have been made to establish a set (Tonetti et al. 2023). The COS does not limit researchers to evaluating only those outcomes; rather, it ensures that these core outcomes are consistently included while still allowing for the collection of additional outcomes tailored to the specific objectives of a given study (Williamson et al. 2017). The present study offers a more recent and targeted effort to define important ClinROs in research on edentulous maxilla rehabilitation.

## 5 Limitations

Among the limitations of the present study, it should be noted that the experts were not randomly selected. While the study included experts from multiple countries, the sample lacks representativeness (Akins et al. 2005). The analysis did not stratify or discuss potential differences in preferences or practices across geographic regions, which could have enriched the interpretation. However, the questionnaire was anonymous, and only information on the geographical distribution of the invited participants and not of the respondents was available. Indeed, in several cases, only a single expert was invited from a given country, and including a question about geographic origin could have risked compromising participant anonymity.

Furthermore, the outcomes are influenced by the threshold set for consensus (Grant et al. 2018), which, as noted earlier, varies across studies (Von Der Gracht 2012). Finally, to reduce experts' burden and avoid the high dropout rates often seen in multiple‐round studies, a pragmatic decision was made to use a single‐round survey (Humphrey‐Murto and de Wit 2019; Quirke et al. 2023). Nonetheless, a response rate of 53.5% was recorded, which may limit the generalizability of the findings and introduce response bias, underscoring the need for cautious interpretation and improved strategies for expert engagement in future studies. This percentage is, however, in line with similar studies using online questionnaires in implant dentistry. In Sanz et al. (2019), only 47.45% of the 138 invited experts responded in the first round, with a further drop to 44.06% in the second round, highlighting the common difficulty of sustaining expert participation in multi‐phase, online survey‐based studies.

## 6 Conclusions

This study explored several aspects of the rehabilitation of the edentulous maxilla through sinus lift and alveolar bone augmentation techniques, collecting expert opinions to guide the development of a consensus and contribute to the creation of clinical practice guidelines for the management of edentulous patients. Key findings were:

CT/CBCT scans were deemed essential for planning, emphasizing precision in anatomical assessment. While minimum subantral bone height for sinus lifts showed variability, experts favored simultaneous implant insertion with 3–4 mm bone height for lateral approaches and 5 mm for crestal. The study also revealed a shift towards guided surgery over freehand techniques for enhanced accuracy, but further comparative studies are needed.

While no formal consensus was reached, most experts routinely prescribed antibiotics. Experts discouraged the use of biologics as a standalone treatment and favored their combination with bone substitutes.

Experts preferred delayed implant placement and loading in complex cases, indicating a cautious approach. For sinus lifts, xenogeneic grafts were favored, and membrane fixation was considered crucial for GBR due to its role in stability and bone regeneration. Consensus was reached for managing Schneiderian membrane perforations up to 10 mm using resorbable membranes, while for larger perforations a more conservative approach, such as interrupting the surgery, was preferred. Soft tissue augmentation at implant placement or second‐stage surgery was strongly supported to establish sufficient keratinized mucosa for peri‐implant health. Regarding provisional restorations, varied clinical preferences were observed.

High implant longevity was anticipated in both native and augmented bone, although more long‐term data are needed for GBR and bone block procedures. The maintenance phase is acknowledged as critical for the long‐term success but without a specific time frame. Routine CBCT is discouraged during the maintenance phase.

The study achieved strong consensus on ClinROs essential for future studies, specifically emphasizing the importance of assessing surgical, prosthetic, and biological complications, along with marginal bone loss. These findings emphasize the critical role of follow‐up visits in monitoring patients undergoing complex procedures, such as those involving sinus lift or bone augmentation. Regular clinical follow‐up would also facilitate the timely identification and management of complications, thereby contributing to long‐term treatment success.

## Author Contributions

**Giulia Brunello:** methodology, software, validation, formal analysis, investigation, data curation, writing – original draft, writing – review and editing. **Franz J. Strauss:** conceptualization, methodology, software, validation, formal analysis, investigation, resources, data curation, writing – original draft, writing – review and editing. **Iva Milinkovic:** writing – original draft, writing – review and editing, investigation. **Ina Kopp:** conceptualization, methodology, data curation, writing – review and editing, supervision. **Frank Schwarz:** writing – review and editing, conceptualization, resources, project administration, supervision. **Hom‐Lay Wang:** conceptualization, resources, project administration, supervision.

## Ethics Statement

The protocol was notified to and approved by the Ethical Committee of the University of Düsseldorf (Protocol no. 2024‐2973).

## Conflicts of Interest

The authors declare no conflicts of interest.

## Supporting information


**Data S1:** clr70018‐sup‐0001‐DataS1.pdf.

**Data S2:** clr70018‐sup‐0002‐DataS2.docx.

## Data Availability

The data that support the findings of this study are available from the corresponding author upon reasonable request.
