# Supplementary material for: 1st Global Consensus for Clinical Guidelines for the Rehabilitation of the Edentulous Maxilla: A Single‐Round Survey on Sinus Lift and Alveolar Bone Augmentation Techniques
Source: Clin Oral Implants Res. 2026 Feb 24;37(Suppl 30):S166–87. doi: 10.1111/clr.70018 (PMC12930134; doi:10.1111/clr.70018)
Supplement: Supplementary file 1 — Data S1: clr70018‐sup‐0001‐DataS1.pdf. [file CLR-37-S166-s001.pdf]

Survey 3 – 1st Global Consensus for Clinical Guidelines 2025

Sinus grafting and alveolar ridge augmentation

* Required

1. If you click on "yes", it means that you consent to participate in the survey, otherwise it ends here. *

- ◯ Yes
- ◯ No

2. Professional specialisation *

Multiple choice possible

- ☐ Periodontology
- ☐ Prosthodontics
- ☐ Oral surgery
- ☐ Oral and maxillofacial surgery
- ☐ None (general practitioner)
- ☐ Other

3. Please specify your professional working environment. *

Multiple choice possible

- ☐ University
- ☐ Public hospital
- ☐ Private clinic
- ☐ Other

4. Which tool(s) should be routinely used for case study of maxillary implant-supported full-arch rehabilitation in combination with sinus lift or other bone grafting procedures? *

Per each answer, please indicate your level of agreement with a score from "strongly disagree" to "strongly agree" ***Make sure to slide the table all the way to the right to see all score levels, including "strongly agree."***

	strongly disagree	disagree	somewhat disagree	neither agree or disagree	somewhat agree	agree	strongly agree
Panoramic radiograph	◯	◯	◯	◯	◯	◯	◯
CT / CBCT	◯	◯	◯	◯	◯	◯	◯
Intraoral photos	◯	◯	◯	◯	◯	◯	◯
Extraoral photos	◯	◯	◯	◯	◯	◯	◯
Facial scanning	◯	◯	◯	◯	◯	◯	◯
Impressions (conventional or intraoral scanning)	◯	◯	◯	◯	◯	◯	◯
Wax-up (conventional or digital)	◯	◯	◯	◯	◯	◯	◯
Mock-up	◯	◯	◯	◯	◯	◯	◯

5. For sinus augmentation, do you consider necessary to investigate by means of CT / CBCT the location and patency of the ostium, the presence of septae as well as the shape and route of the intrabony canal of the superior posterior alveolar artery? *

Per each answer, please indicate your level of agreement with a score from "strongly disagree" to "strongly agree" ***Make sure to slide the table all the way to the right to see all score levels, including "strongly agree."***

	strongly disagree	disagree	somewhat disagree	neither agree or disagree	somewhat agree	agree	strongly agree
Lateral sinus lift	◯	◯	◯	◯	◯	◯	◯
Crestal sinus lift	◯	◯	◯	◯	◯	◯	◯

6. In presence of adequate ridge width, which do you consider the minimum subantral bone height (in mm) to perform:
Lateral sinus lift and simultaneous implant placement *

The value must be a number

7. In presence of adequate ridge width, which do you consider the minimum subantral bone height (in mm) to perform:
Crestal sinus lift and simultaneous implant placement *

The value must be a number

8. In case of multiple implant placement in fully edentulous maxilla, should freehand surgery be preferred over static/dynamic guided surgery?
*

Per each answer, please indicate your level of agreement with a score from "strongly disagree" to "strongly agree" ***Make sure to slide the table all the way to the right to see all score levels, including "strongly agree."***

	strongly disagree	disagree	somewhat disagree	neither agree or disagree	somewhat agree	agree	strongly agree
Bone grafting and simultaneous implant placement	◯	◯	◯	◯	◯	◯	◯
Lateral sinus lift and simultaneous implant placement	◯	◯	◯	◯	◯	◯	◯
Crestal sinus lift and simultaneous implant placement	◯	◯	◯	◯	◯	◯	◯
Delayed implant placement after bone grafting/sinus lift	◯	◯	◯	◯	◯	◯	◯

9. In the posterior maxilla in presence of terminal dentition in the posterior sector and inadequate residual bone requiring sinus lift/bone grafting, which protocol do you prefer among immediate, early and delayed implant placement? *

◯ Immediate implant placement

◯ Early implant placement

◯ Delayed implant placement

10. Is immediate loading protocol (within 1 week from implant placement) a secure treatment option in case of simultaneous sinus lift/bone grafting and good primary stability? *

Per each answer, please indicate your level of agreement with a score from "strongly disagree" to "strongly agree" ***Make sure to slide the table all the way to the right to see all score levels, including "strongly agree."***

	strongly disagree	disagree	somewhat disagree	neither agree or disagree	somewhat agree	agree	strongly agree
Splinted provisional restoration	◯	◯	◯	◯	◯	◯	◯
Non-splinted provisional restoration	◯	◯	◯	◯	◯	◯	◯

11. In case of primary bone augmentation or lateral sinus lift and delayed implant placement how much time do you usually wait before implant placement? *

Please indicate the time interval in months

The value must be a number

12. Which bone substitute material(s) do you preferably use among autologous, allogeneic, xenogeneic, synthetic ones or a combination of various materials depending on the chosen surgical approach? *

Per each answer, please indicate one option. ***Make sure to slide the table all the way to the right to see all 5 options***

	Autologous	Allogeneic	Xenogeneic	Synthetic	Combination
Bone grafting and simultaneous implant placement	◯	◯	◯	◯	◯
Lateral sinus lift and simultaneous implant placement	◯	◯	◯	◯	◯
Crestal sinus lift and simultaneous implant placement	◯	◯	◯	◯	◯
Bone grafting/sinus lift and delayed implant placement	◯	◯	◯	◯	◯

13. In case of GBR for vertical bone augmentation, should the membrane always be fixed (e.g. with pins, suture, etc.)? *

Please indicate your level of agreement with a score from "strongly disagree" to "strongly agree" ***Make sure to slide the table all the way to the right to see all score levels, including "strongly agree."***

	strongly disagree	disagree	somewhat disagree	neither agree or disagree	somewhat agree	agree	strongly agree
Agreement	◯	◯	◯	◯	◯	◯	◯

14. In your opinion, in case of GBR, does the membrane exposure have a detrimental influence on the outcome of bone regeneration? *

Per each answer, please indicate your level of agreement with a score from "strongly disagree" to "strongly agree" ***Make sure to slide the table all the way to the right to see all score levels, including "strongly agree."***

	strongly disagree	disagree	somewhat disagree	neither agree or disagree	somewhat agree	agree	strongly agree
Resorbable membrane (collagen)	◯	◯	◯	◯	◯	◯	◯
Non-resorbable membrane (e.g. dPTFE)	◯	◯	◯	◯	◯	◯	◯
Titanium mesh	◯	◯	◯	◯	◯	◯	◯

15. In case of primary bone augmentation what type of provisional restauration do you prefer during the healing period in the fully edentulous ridge? *

16. Do you consider biologics (e.g. blood concentrates) as appropriate treatment options for the following indications? *

Per each answer, please indicate your level of agreement with a score from "strongly disagree" to "strongly agree" ***Make sure to slide the table all the way to the right to see all score levels, including "strongly agree."***

	strongly disagree	disagree	somewhat disagree	neither agree or disagree	somewhat agree	agree	strongly agree
Alone without bone substitutes for sinus lifting	◯	◯	◯	◯	◯	◯	◯
Alone in the extraction socket	◯	◯	◯	◯	◯	◯	◯
Combined with bone substitutes	◯	◯	◯	◯	◯	◯	◯
As a barrier membrane in GBR	◯	◯	◯	◯	◯	◯	◯

17. Do you routinely prescribe antibiotics as prophylaxis during multiple implant placement for the rehabilitation of the fully edentulous maxilla? *

◯ Always

◯ Yes, in the following circumstances listed below

◯ Never

18. Please indicate which circumstances *

Multiple selections possible

- [ ] Medically compromised patients (e.g. risk of infective endocarditis, immunocompromised, diabetes, etc.)
- [ ] Lateral sinus lift
- [ ] Crestal sinus lift
- [ ] Lateral sinus lift only in presence of sinus infection
- [ ] Crestal sinus lift only in presence of sinus infection
- [ ] Guided bone regeneration (GBR
- [ ] Bone blocks
- [ ] Immediate implant placement
- [ ] Immediate implant placement only if involving infected sockets
- [ ] Other

19. Do you routinely prescribe antibiotics as prophylaxis after multiple implant placement for the rehabilitation of the fully edentulous maxilla? *

- ( ) Always
- ( ) Yes, in the following circumstances listed below
- ( ) Never

20. Please indicate which circumstances *

Multiple selections possible

- [ ] Medically compromised patients (e.g. risk of infective endocarditis, immunocompromised, diabetes, etc.)
- [ ] Lateral sinus lift
- [ ] Crestal sinus lift
- [ ] Lateral sinus lift only in presence of sinus infection
- [ ] Crestal sinus lift only in presence of sinus infection
- [ ] Guided bone regeneration (GBR
- [ ] Bone blocks
- [ ] Immediate implant placement
- [ ] Immediate implant placement only if involving infected sockets
- [ ] Other

21. In case of "yes", please specify for how many days.

____

The value must be a number

22. In absence of complications, are the following procedures justified at least once a year in the long-term follow-up? *

Per each answer, please indicate your level of agreement with a score from "strongly disagree" to "strongly agree" ***Make sure to slide the table all the way to the right to see all score levels, including "strongly agree."***

	strongly disagree	disagree	somewhat disagree	neither agree or disagree	somewhat agree	agree	strongly agree
Full-mouth six-point pocket chart	◯	◯	◯	◯	◯	◯	◯
Intraoral radiographs	◯	◯	◯	◯	◯	◯	◯
Panoramic radiographs	◯	◯	◯	◯	◯	◯	◯
CT/CBCT	◯	◯	◯	◯	◯	◯	◯

23. Do you think that the following materials / techniques will be commonly applied within the next five years for the rehabilitation of the fully edentulous maxilla with dental implants and regenerative techniques? *

Per each answer, please indicate your level of agreement with a score from "strongly disagree" to "strongly agree" ***Make sure to slide the table all the way to the right to see all score levels, including "strongly agree."***

	strongly disagree	disagree	somewhat disagree	neither agree or disagree	somewhat agree	agree	strongly agree
Dynamic computer-guided surgery	◯	◯	◯	◯	◯	◯	◯
Robotic-guided surgery	◯	◯	◯	◯	◯	◯	◯
3D printed bone scaffold	◯	◯	◯	◯	◯	◯	◯
3D printed meshes	◯	◯	◯	◯	◯	◯	◯
Stem cells	◯	◯	◯	◯	◯	◯	◯

24. In case of perforation of the Schneiderian membrane during lateral sinus lift, to what extent do you consider the following options suitable?
Perforation up to 5 mm *

Per each answer, please indicate your level of agreement with a score from "strongly disagree" to "strongly agree" ***Make sure to slide the table all the way to the right to see all score levels, including "strongly agree."***

	strongly disagree	disagree	somewhat disagree	neither agree or disagree	somewhat agree	agree	strongly agree
Nothing, I interrupt the surgery	◯	◯	◯	◯	◯	◯	◯
Post-perforation clot formation	◯	◯	◯	◯	◯	◯	◯
Suturing of the Schneiderian membran	◯	◯	◯	◯	◯	◯	◯
Collagen membrane	◯	◯	◯	◯	◯	◯	◯
Blood concentrates (e.g. PRF)	◯	◯	◯	◯	◯	◯	◯
Haemostatic agents	◯	◯	◯	◯	◯	◯	◯
Bone blocks	◯	◯	◯	◯	◯	◯	◯

25. In case of perforation of the Schneiderian membrane during lateral sinus lift, to what extent do you consider the following options suitable?
Perforation between 5 and 10 mm *

Per each answer, please indicate your level of agreement with a score from "strongly disagree" to "strongly agree" ***Make sure to slide the table all the way to the right to see all score levels, including "strongly agree."***

	strongly disagree	disagree	somewhat disagree	neither agree or disagree	somewhat agree	agree	strongly agree
Nothing, I interrupt the surgery	◯	◯	◯	◯	◯	◯	◯
Post-perforation clot formation	◯	◯	◯	◯	◯	◯	◯
Suturing of the Schneiderian membran	◯	◯	◯	◯	◯	◯	◯
Collagen membrane	◯	◯	◯	◯	◯	◯	◯
Blood concentrates (e.g. PRF)	◯	◯	◯	◯	◯	◯	◯
Haemostatic agents	◯	◯	◯	◯	◯	◯	◯
Bone blocks	◯	◯	◯	◯	◯	◯	◯

26. In case of perforation of the Schneiderian membrane during lateral sinus lift, to what extent do you consider the following options suitable?
Perforation greater than 10 mm *

Per each answer, please indicate your level of agreement with a score from "strongly disagree" to "strongly agree" ***Make sure to slide the table all the way to the right to see all score levels, including "strongly agree."***

	strongly disagree	disagree	somewhat disagree	neither agree or disagree	somewhat agree	agree	strongly agree
Nothing, I interrupt the surgery	◯	◯	◯	◯	◯	◯	◯
Post-perforation clot formation	◯	◯	◯	◯	◯	◯	◯
Suturing of the Schneiderian membran	◯	◯	◯	◯	◯	◯	◯
Collagen membrane	◯	◯	◯	◯	◯	◯	◯
Blood concentrates (e.g. PRF)	◯	◯	◯	◯	◯	◯	◯
Haemostatic agents	◯	◯	◯	◯	◯	◯	◯
Bone blocks	◯	◯	◯	◯	◯	◯	◯

27. In case of lack of keratinized tissue, when do you generally perform soft tissue augmentation to establish keratinized mucosa around dental implants? *

◯ At the time of implant insertion / second stage surgery

◯ After loading for prevention of complications

◯ In case of complications

◯ Never

28. Under normal circumstances, how long do you estimate the longevity of the implant treatment without complications in the following cases? *

Per each answer, please indicate one option

	less than 5 years	less than 10 years	more than 10 years
In native bone	◯	◯	◯
Lateral sinus lift	◯	◯	◯
Crestal sinus lift	◯	◯	◯
GBR	◯	◯	◯
Bone blocks	◯	◯	◯

29. In case of maxillary full-arch rehabilitation with dental implants requiring sinus grafting/ alveolar ridge augmentation, do the following factors affect the overall patient satisfaction in the short term? *

Per each answer, please indicate your level of agreement with a score from "strongly disagree" to "strongly agree" ***Make sure to slide the table all the way to the right to see all score levels, including "strongly agree."***

	strongly disagree	disagree	somewhat disagree	neither agree or disagree	somewhat agree	agree	strongly agree
Aesthetics	◯	◯	◯	◯	◯	◯	◯
Chewing function	◯	◯	◯	◯	◯	◯	◯
Type of definitive prosthesis (i.e. fixed vs removable	◯	◯	◯	◯	◯	◯	◯
Phonetic	◯	◯	◯	◯	◯	◯	◯
Easy of cleaning	◯	◯	◯	◯	◯	◯	◯
Preoperative dental status	◯	◯	◯	◯	◯	◯	◯
Discomfort with the surgery	◯	◯	◯	◯	◯	◯	◯
Invasiveness of the procedure	◯	◯	◯	◯	◯	◯	◯
Number of surgeries	◯	◯	◯	◯	◯	◯	◯
Complications	◯	◯	◯	◯	◯	◯	◯
Overall treatment time	◯	◯	◯	◯	◯	◯	◯
Duration of the surgery	◯	◯	◯	◯	◯	◯	◯
Loading protocol	◯	◯	◯	◯	◯	◯	◯
Pain	◯	◯	◯	◯	◯	◯	◯
Need for medications	◯	◯	◯	◯	◯	◯	◯
Immediate post-surgery prosthesis	◯	◯	◯	◯	◯	◯	◯
Costs	◯	◯	◯	◯	◯	◯	◯

30. When you choose a procedure, on what do you base your decision? *

- ◯ Ease of treatment
- ◯ Evidence
- ◯ Both

31. How important is the difficulty of the procedure when you choose one? *

Please indicate your level of agreement with a score from "strongly disagree" to "strongly agree" ***Make sure to slide the table all the way to the right to see all score levels, including "strongly agree."***

	strongly disagree	disagree	somewhat disagree	neither agree or disagree	somewhat agree	agree	strongly agree
Agreement	◯	◯	◯	◯	◯	◯	◯

32. In future studies on maxillary full-arch rehabilitation with dental implants, do you consider relevant the following patient reported outcome measures (PROMs)? *

Per each answer, please indicate your level of agreement with a score from "strongly disagree" to "strongly agree" ***Make sure to slide the table all the way to the right to see all score levels, including "strongly agree."***

	strongly disagree	disagree	somewhat disagree	neither agree or disagree	somewhat agree	agree	strongly agree
Quality of masticatory function questionnaire (QMFQ)	◯	◯	◯	◯	◯	◯	◯
Oral health impact profile for edentulous people (OHIP-EDENT)	◯	◯	◯	◯	◯	◯	◯
Visual analogue scale (VAS) for satisfaction	◯	◯	◯	◯	◯	◯	◯
Chewing ability questionnaire	◯	◯	◯	◯	◯	◯	◯
Denture satisfaction questionnaire	◯	◯	◯	◯	◯	◯	◯
Micro aesthetics questionnaire (from smile close up photos	◯	◯	◯	◯	◯	◯	◯
Macro aesthetics questionnaire (from smile portrait photos)	◯	◯	◯	◯	◯	◯	◯

33. In future studies on maxillary full-arch rehabilitation with dental implants, do you consider relevant the following clinician-reported outcome measures (CROMs)? *

Per each answer, please indicate your level of agreement with a score from "strongly disagree" to "strongly agree" ***Make sure to slide the table all the way to the right to see all score levels, including "strongly agree."***

	strongly disagree	disagree	somewhat disagree	neither agree or disagree	somewhat agree	agree	strongly agree
Implant survival - Vertical bone height assessed on CBCT	○	○	○	○	○	○	○
Surgical complications	○	○	○	○	○	○	○
Prosthetic complications	○	○	○	○	○	○	○
Marginal bone loss (MBL)	○	○	○	○	○	○	○
Resonance frequency analysis	○	○	○	○	○	○	○
Implant primary stability	○	○	○	○	○	○	○
Plaque score	○	○	○	○	○	○	○
Peri-implant mucositis and Peri-implantitis	○	○	○	○	○	○	○
Duration of the surgery	○	○	○	○	○	○	○
Ridge Width Measurement	○	○	○	○	○	○	○
Ridge Height Measurement	○	○	○	○	○	○	○
Keratinized Tissue Width	○	○	○	○	○	○	○
Clinician's Assessment of Treatment Success	○	○	○	○	○	○	○
Recommendations for Future Procedures	○	○	○	○	○	○	○
Surgery Difficulty	○	○	○	○	○	○	○

This content is neither created nor endorsed by Microsoft. The data you submit will be sent to the form owner.

Microsoft Forms
