# Supplementary material for: 1st Global Consensus for Clinical Guidelines for the Rehabilitation of the Edentulous Maxilla: A Single‐Round Survey on Sinus Lift and Alveolar Bone Augmentation Techniques
Source: Clin Oral Implants Res. 2026 Feb 24;37(Suppl 30):S166–87. doi: 10.1111/clr.70018 (PMC12930134; doi:10.1111/clr.70018)
Supplement: Supplementary file 2 — Data S2: clr70018‐sup‐0002‐DataS2.docx. [file CLR-37-S166-s002.docx]

**SUPPLEMENTARY FIGURES**

For each 7-point Likert scale question, a graph was created reporting medians and interquartile ranges (IQR).

**Suppl. Figure 1. Which tool(s) should be routinely used for case study of maxillary implant-supported full-arch rehabilitation in combination with sinus lift or other bone grafting procedures?**

**Suppl. Figure 2. For sinus augmentation, do you consider necessary to investigate by means of CT / CBCT the location and patency of the ostium, the presence of septae as well as the shape and route of the intrabony canal of the superior posterior alveolar artery?**

**Suppl. Figure 3. In case of multiple implant placement in fully edentulous maxilla, should freehand surgery be preferred over static/dynamic guided surgery?**

**Suppl. Figure 4. Is immediate loading protocol (within 1 week from implant placement) a secure treatment option in case of simultaneous sinus lift/bone grafting and good primary stability?**

**Suppl. Figure 5. In case of GBR for vertical bone augmentation, should the membrane always be fixed (e.g. with pins, suture, etc.)?**

**Suppl. Figure 6. In your opinion, in case of GBR, does the membrane exposure have a detrimental influence on the outcome of bone regeneration?**

**Suppl. Figure 7. Do you consider biologics (e.g. blood concentrates) as appropriate treatment options for the following indications?**

**Suppl. Figure 8. In absence of complications, are the following procedures justified at least once a year in the long-term follow-up?**

**
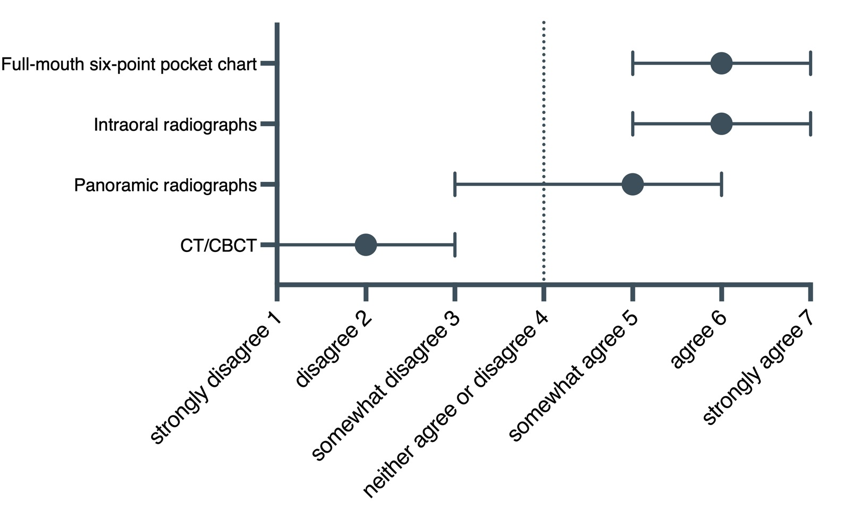
**

**Suppl. Figure 9. Do you think that the following materials / techniques will be commonly applied within the next five years for the rehabilitation of the fully edentulous maxilla with dental implants and regenerative techniques?**


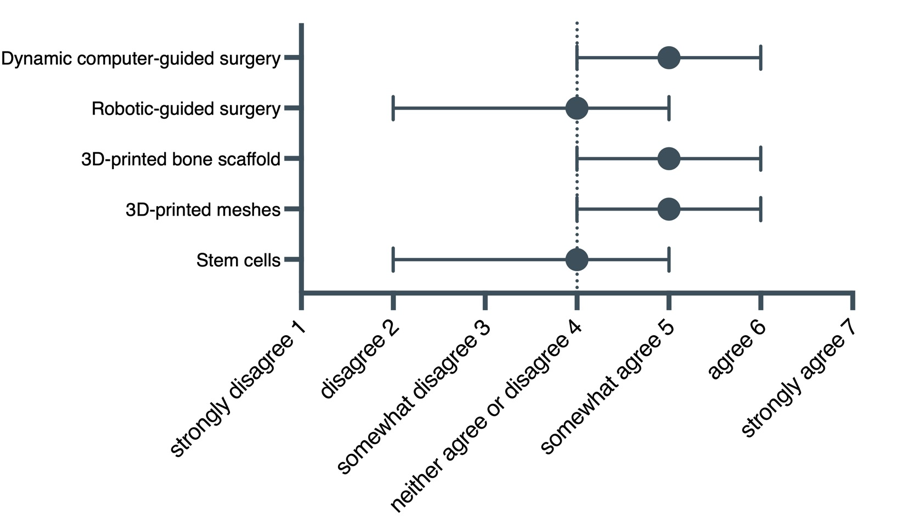


**Suppl. Figure 10. In case of perforation of the Schneiderian membrane during lateral sinus lift, to what extent do you consider the following options suitable? Perforation up to 5 mm.**

**Suppl. Figure 11. In case of perforation of the Schneiderian membrane during lateral sinus lift, to what extent do you consider the following options suitable? Perforation between 5 and 10 mm.**

**Suppl. Figure 12. In case of perforation of the Schneiderian membrane during lateral sinus lift, to what extent do you consider the following options suitable? Perforation greater than 10 mm.**

**Suppl. Figure 13. In case of maxillary full-arch rehabilitation with dental implants requiring sinus grafting/ alveolar ridge augmentation, do the following factors affect the overall patient satisfaction in the short term?**

**
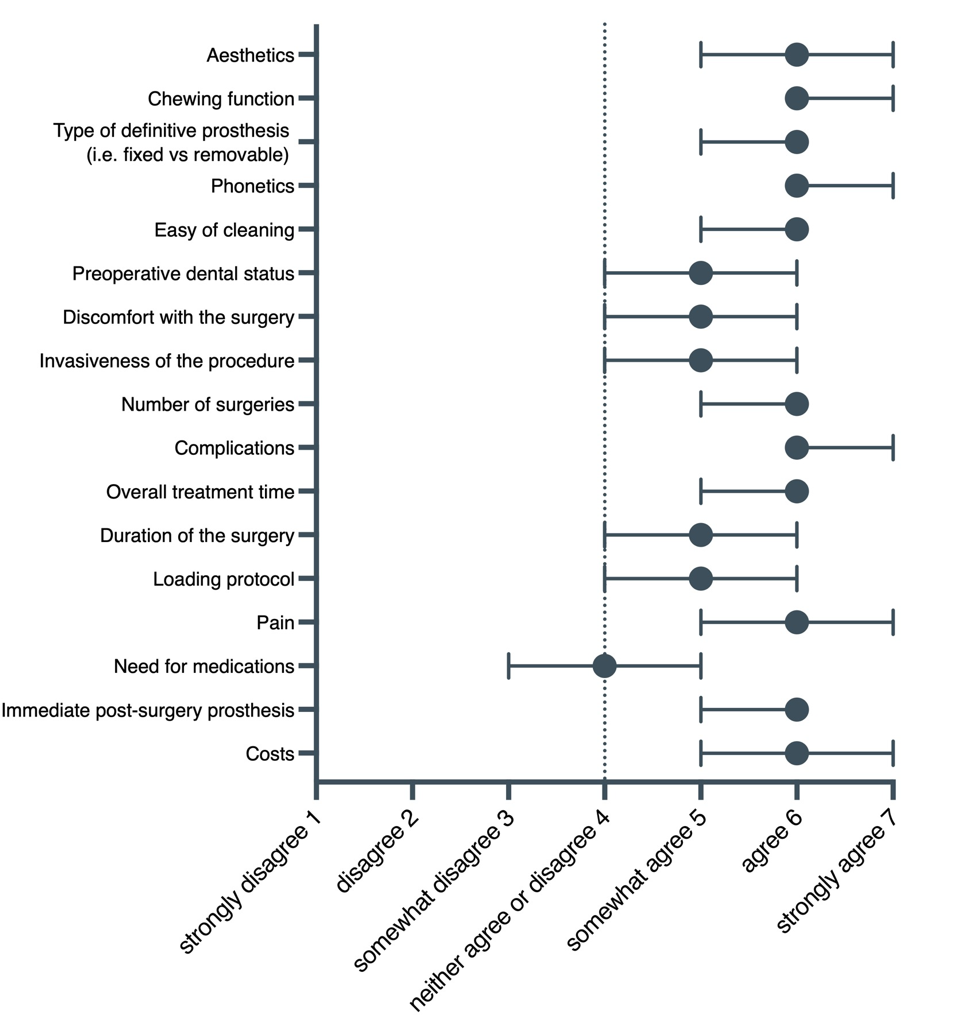
**

**Suppl. Figure 14. How important is the difficulty of the procedure when you choose one?**

**Suppl. Figure 15. In future studies on maxillary full-arch rehabilitation with dental implants, do you consider relevant the following patient-reported outcome measures (PROMs)?**

**
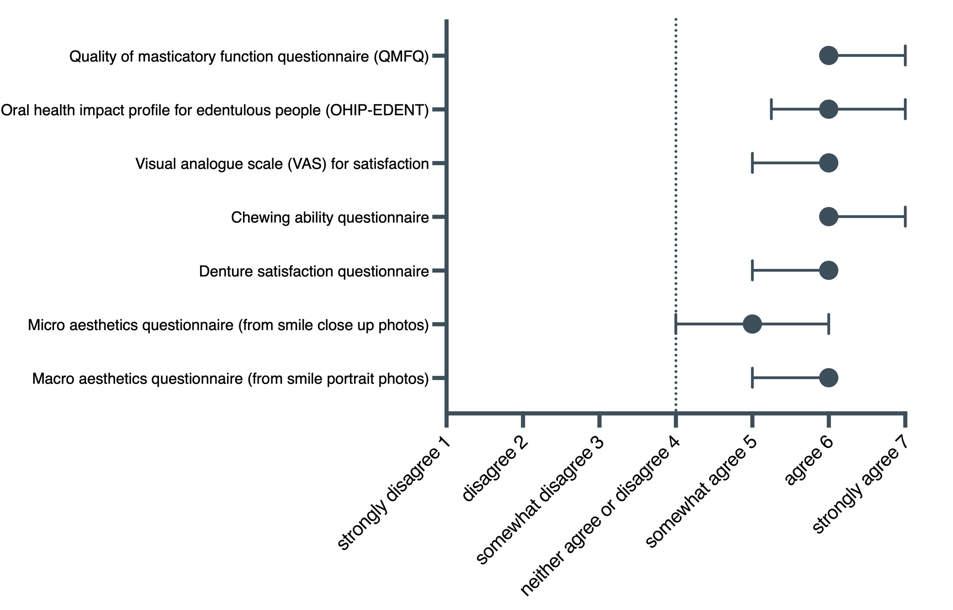
**

**Suppl. Figure 16. In future studies on maxillary full-arch rehabilitation with dental implants, do you consider relevant the following clinician-reported outcome measures (ClinROMs)?**

**
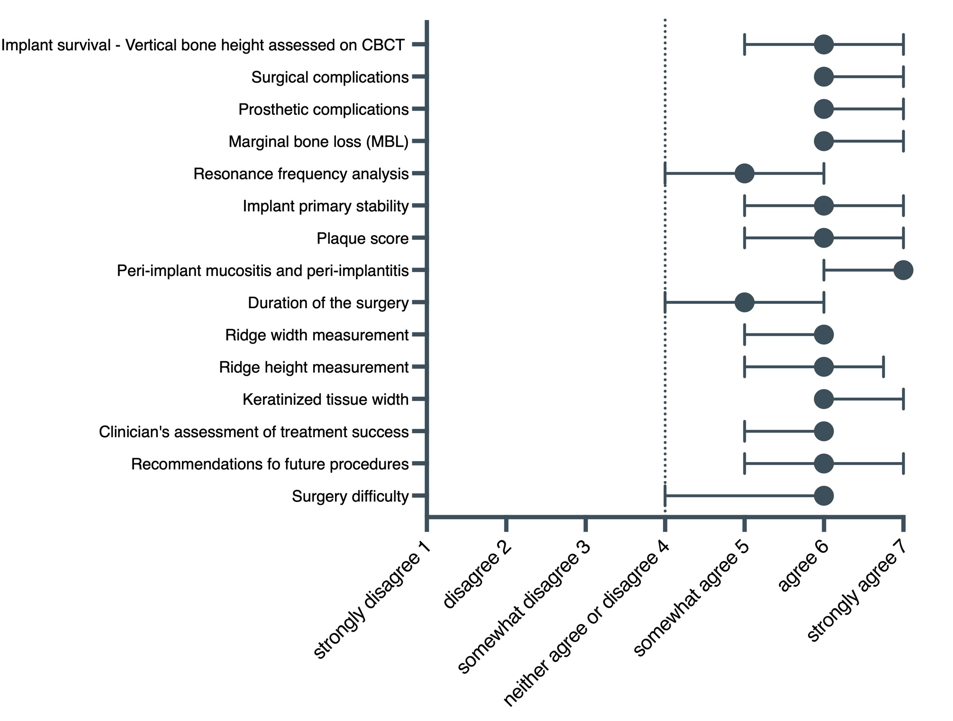
**
